# Substantial differences occur between canopy and ambient climate: Quantification of interactions in a greenhouse-canopy system

**DOI:** 10.1371/journal.pone.0233210

**Published:** 2020-05-29

**Authors:** A. van Westreenen, N. Zhang, J. C. Douma, J. B. Evers, N. P. R. Anten, L. F. M. Marcelis

**Affiliations:** 1 Horticulture and Product Physiology Group, Wageningen University and Research, Wageningen, The Netherlands; 2 Centre for Crop System Analysis, Wageningen University and Research, Wageningen, The Netherlands; 3 Laboratory of Entomology, Wageningen University and Research, Wageningen, The Netherlands; Huazhong Agriculture University, CHINA

## Abstract

Organ temperature and variation therein plays a key role in plant functioning and its responses to e.g. climate change. There is a strong feedback between organ, especially leaf, temperature and the climate within the canopy (canopy climate), which in turn interacts with the climate outside the canopy (ambient climate). For greenhouses, the determinants of this interplay and how they drive differences between canopy and ambient climate are poorly understood. Yet, as many experiments on both regular greenhouse crops and field crops are done in greenhouses, this is crucial to know. Therefore, we designed an experiment to quantify the differences between ambient and canopy climate and leaf temperature. A path analysis was performed to quantify the interactions between components of the greenhouse canopy-climate system. We found that with high radiation the canopy climate can be up to 5°C cooler than the ambient climate, while for cloudy days this was only 2°C. Canopy relative humidity (*RH*) was up to 25% higher compared to ambient *RH*. We showed that radiation is very important for these climate differences, but that this effect could be partly counteracted by turning off supplementary light (i.e. due to its indirect effects e.g. changing light distribution). Leaf temperature was substantially different, both higher and lower, from the canopy air temperature. This difference was determined by leaf area index (*LAI*), temperature of the heating pipe and the use of supplementary light, which all strongly influence radiation, either shortwave or thermal radiation. The difference between leaf and ambient air temperature could be decreased by decreasing the *LAI* or increasing the temperature of the heating pipe.

## Introduction

Organ temperature and variation therein plays a key role in plant functioning and its responses to e.g. climate change. First, organ temperature directly affects photosynthetic carbon gain and organ water losses. Second, the organ temperature drives plant development. Third, the development and activity of diseases and pests is strongly determined by organ temperature and local humidity including dew formation [1]. The organ temperature can substantially deviate from air temperature (e.g. apex temperature in [2]) and strongly depends on environmental conditions [3]. This dependence can introduce substantial variability between repetitions and experiments due to weather changes (e.g. between seasons and/or years). Since in the greenhouse the climate can at least partly be controlled, many experiments on both wild and domesticated (crop) plants are carried out in greenhouses [4]. However, in most experiments focussed at plant growth and development, variation in organ temperature and its difference with greenhouse climate is not considered. This may lead to important errors or misinterpretations. For example, in estimates of temperature sums of developmental stages, temperature relationships of physiological processes or organ-level humidity and wetness and associated pest and disease outbreaks.

The organ temperature strongly depends on the microclimate. In the literature, different temporarily and spatially small-scale climates are all described by the term microclimate (e.g. [5, 6]). Therefore, we define two climates with different spatial and temporal scales, (1.) the ambient climate, and (2.) the canopy climate. We define climate as the prevailing environmental conditions in a specific area. First, the ambient climate, i.e. the climate outside the canopy, has the largest spatio-temporal scale of the two. In the field this is referred to as the weather, while in a greenhouse it is the climate within the greenhouse outside of the canopy. The ambient climate is influenced by the outside weather, the climate control system and the canopy (e.g. by transpiration). Second, the canopy climate refers to the climatic conditions within the canopy. The canopy climate can be highly heterogeneous in space [7] and time, and may depend on canopy structure e.g. the leaf area index (*LAI*), leaf angle distribution, which determines radiation distribution, and height of the stand. For instance, doubling tomato plant densities increased the difference between ambient and canopy air temperature with 1°C [8], probably due to larger gradients in light interception and less air exchange between ambient and canopy air. Next to through plant architectural traits, the canopy modifies its climate through transpiration. As such, the canopy climate can be very different from the ambient climate. This has been extensively studied for field crops like corn (e.g. [9]), wheat (e.g. [10]) and soybeans [11] where the day-time canopy air temperature has been found to be 2°C cooler than ambient temperatures, although the difference was strongly affected by water-stress. Little observations on temperature and humidity gradients in the canopy have been done on greenhouse crops.

In the greenhouse there are many possibilities to control the climate, e.g. by heating, ventilation and humidification. With these climate measures the differences between ambient and canopy climate can be modified. Canopy air temperature can be up to 5°C cooler than the ambient temperature with a cooling system below the gutters [7], although this difference is strongly influenced by the location of the cooling system [12]. Furthermore, the heating system and ventilation influence the temperature gradient by adding heat [13] or by variable proportion of mixing the heat and moisture over the ambient and canopy air [14]. The same holds for humidity profiles; humidity is higher inside the canopy than the ambient climate in regular tomato greenhouses [5]. It is not clear how the difference between ambient and canopy climate changes during the seasons, although this difference is crucially important for the organ temperature in determining plant, pest and disease development [15].

There is some literature describing the canopy climate inside greenhouses (e.g. [5, 7]) and how this is influenced by growing plants [8] and different climate measures (e.g. [12, 13]). However, it is not clear how the canopy climate is determined by the interaction between these greenhouse components. As noted above, quantifying the canopy climate and the organ temperature is essential to understand plant gas exchange, plant growth, plant development, and the development of pests and pathogens. Therefore, in this paper we aim to quantify the relative importance of the ambient temperature and humidity, as affected by climate control measures and the developing canopy (i.e. different leaf area index, *LAI*) for 1.) the canopy air temperature and humidity, and 2.) the organ temperature, where we focus on leaf temperature in this study. To this end, we designed an experiment to quantify the differences between the ambient and canopy climate and leaf temperature. A path analysis was performed to quantify the interactions between the components of the greenhouse-canopy-climate system.

## Materials and methods

### Experimental set-up

To quantify the difference between the different climates and the relationships between them, an experiment was conducted in one 12 x 12m compartment in a Venlo-type glasshouse with 4.75m height at the roof gutter in Wageningen, The Netherlands (51.989N, 5.664E). The compartment contained six beds consisting of two gutters each. On these gutters rooted rose-cuttings (*rosa* x *hybrida*, cv. Red Naomi!) were planted in a zigzag pattern on the rockwool slabs at a plant density of 7.5 plants m^−2^ to closely resemble a realistic commercial set-up. The crop was watered with drip irrigation that also provided the nutrients, which were both adjusted according to the plant status. The ambient climate was set per growing cycle to get the best growing conditions within the time of the year and were common practice for plants like roses. The conditions were set in consultation with a crop advisor. The measurements were done from May 2, 2017 till June 14, 2017. Greenhouse day and night temperature were set to 19°C and 17°C respectively, which was mainly controlled by the windows (natural ventilation) and the heating pipes below the canopy. Supplementary lights (positioned in a checkerboard pattern approximately 3m above the top of the canopy; 600W high pressure sodium, Philips, Eindhoven, The Netherlands) with narrow beam reflectors were turned on when outside global radiation dropped below 200W m^−2^ and turned off when it increased above 300W m^−2^. The intensity of the supplementary light at the top of the canopy was around 150*μ*mol m^−2^ s^−1^. The shading screens were closed when outside global radiation increased above 600W m^−2^ and opened when it dropped below 500W m^−2^.

Two weeks after planting, the flower buds were removed and the shoots were bent such that the shoot tips pointed slightly downwards, following commercial cut-rose production practices. The bent shoots provide energy for the upright shoots that will be harvested. To create a fully bent canopy from the start of the experiment, the next shoots that appeared were also bent after removal of the flower bud. During the rest of the experiment, all upright shoots were harvested at the same day by pruning at the first 5-leaflet leaf when 80% of the shoots were flowering. Subsequently, this leaf was removed to stimulate axillary bud break. Shoots that were thinner than 5mm were bent in order to keep a full bent canopy. Any lateral branches on the upright shoots were removed. Plants had on average two upright shoots, resulting in an *LAI* of the upright canopy of 1.6.

Pests were prevented with biological control, and in some accidental cases sprayed with insecticides. To prevent disease development, the fungicide Meltatox was sprayed after every harvest. During the growing cycle (i.e. from one harvest to the next), Meltatox was sprayed locally in case of a sudden powdery mildew outbreak. Meltatox was applied with a dosage of 250mL 100L^−1^, All data used in the analysis were taken at least 48 hours after spraying.

### Climatic measurements

**Fig 1:**
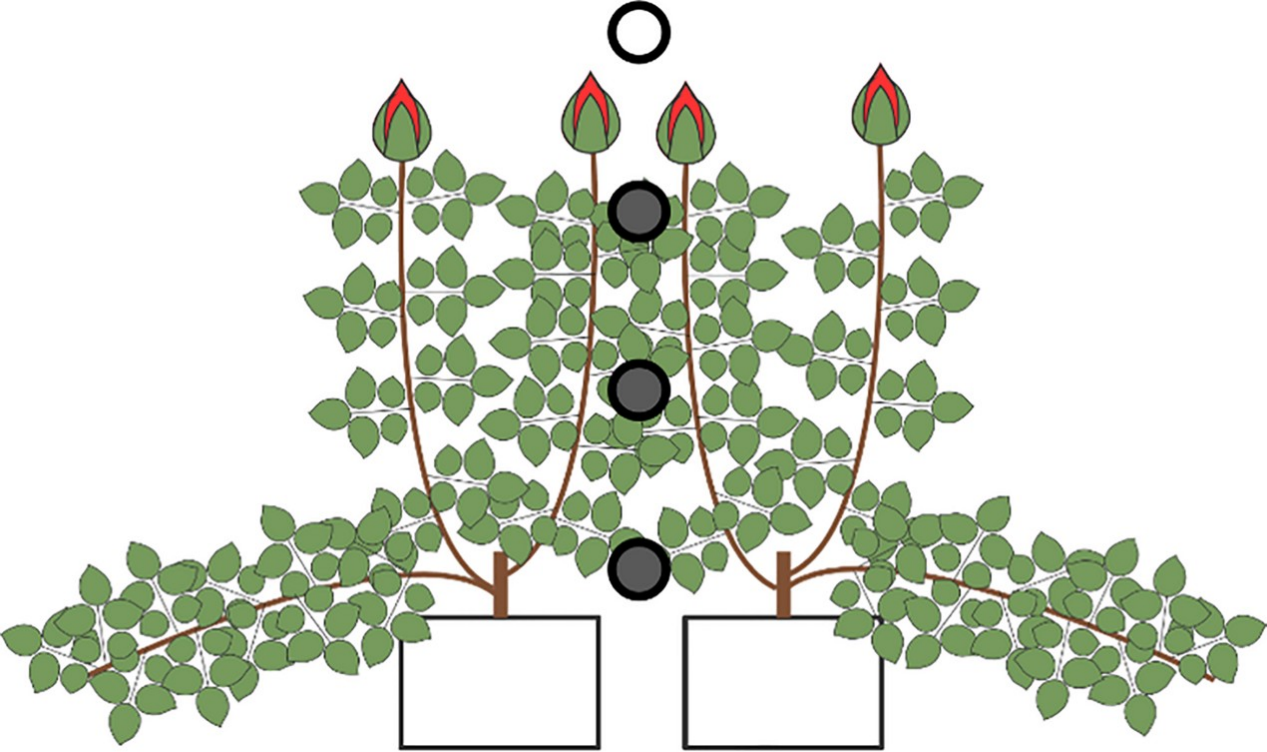
Set-up of the canopy climate measurements. The open circle refers to the reference sensor and the climate box that measures the ambient climate. The filled circles indicate the location of *PAR*, temperature and relative humidity measurements. The top sensor was positioned at a height of 1.8m from the ground, which was just above the canopy when the canopy was fully developed. The other sensors were placed at an interval of 0.3m.

In this experiment, we quantified the ambient, canopy climate and the leaf temperature. The ambient climate was measured with a standard ventilated greenhouse climate box (Hoogendoorn, Vlaardingen, The Netherlands; open circle in Fig 1) that collected climate data, i.e. CO_2_ concentration (EE820, E+E Elektronik, Germany), temperature and relative humidity (*RH*; dry and wet bulb temperature with a Pt500 sensor); all at a five-minute interval. At the same frequency, outside global radiation (250-3000 nm; SR11, Hukseflux, Delft, The Netherlands), window opening, temperature of the heating pipe, shading and supplementary lighting were logged by the climate computer. Greenhouse incoming shortwave (300—2800 nm) and longwave radiation (4.5—42 *μ*m; CNR4, Kipp&Zonen B.V., Delft, The Netherlands) was measured 1.5m above the top of the canopy and 1.5m below the assimilation lights at a measurement frequency of 1 measurement per minute.

The canopy climate was quantified by measuring vertical profiles of photosynthetically active radiation (*PAR*; 400-700 nm) (LI-190SA; LI-COR, USA), temperature and *RH* (the latter two using Hygroclip HC2; Rotronic, Switzerland). These measurements were done in the upright canopy (filled circles in Fig 1). Measurements of the canopy climate were logged with a 1 s interval, enabling us to also study effects of rapid fluctuations inside the greenhouse on canopy climate. Sensors were positioned at a fixed position during the experiment such that the top sensor at 1.8 m from the ground, which was measuring the ambient climate, was just above the canopy when the canopy was fully developed. The other sensors were placed at 0.3 m intervals going down into the canopy, such that the lowest sensor was 0.9 m below the top sensor.

The leaf surface temperature was measured with a K-type thermocouple (LabFaculty, UK). At the leaf surface the humidity was assumed to be 100% [16]. Leaf temperature was measured on two fully unfolded leaves per layer (i.e. on the same height as the *PAR* sensors) resulting in six leaves being measured. The thermocouples were positioned at the abaxial side (to prevent heating of the sensor by direct sunlight) in the middle of the terminal leaflet and were checked every 24 hours for their touching of the leaf. If the thermocouples were found to not be touching the leaf, the data of the 24 hours before were discarded. For the comparison between different weather types four or five days of each weather type were averaged to prevent looking at single events. The comparison is done for the difference between ambient and canopy air temperature, relative humidity (*RH*) and leaf temperature.

### Estimation of LAI

At the end of five growth cycles (S1 Table), the area of individual leaves (sample size 1406) was measured with a LI-3100C (LI-COR, USA) on in total 118 mature upright shoots when the flower bud was open. Additionally, we measured leaf length and width, and the number of leaflets for the individual leaves. These measurements were used to calibrate an allometric relation for the area of individual leaves [17] to estimate the area of the leaves weekly:

$$\begin{array}{c} \sqrt{LA_{k}}=a+bL+cW+d\sqrt{LW}+eN_{ll}+fLN_{ll}+gWN_{ll}+h\sqrt{LW}N_{ll} \end{array}$$ (1)

where *LA*_*k*_ is the leaf area [m^2^] of a leaf at rank *k*, *L* and *W* are the length and width of a leaf [m] respectively, *N*_*ll*_ is the number of leaflets on a leaf and *a* − *h* are regression coefficients.

Calibration of the allometric relation resulted in the following regression coefficients: 0.597, 0.704, 0.942, -1.071, -0.118, -0.030, -0.057 and 0.109 for coefficient *a* − *h* respectively. Validating this relation with destructive measurements of developing shoots in the last growth cycle yielded a model with an R^2^ of 0.92 and a relative root-mean-square error (RMSE) of 0.025. This allometric relation was then used to calculate the leaf area of leaves of 16 to 50 plants (depending on the growth cycle; S1 Table) where we measured leaf length and width, and the number of leaflets non-destructively every week.

The total leaf area of a shoot was calculated by summing the leaf area of individual leaves. Based on the number of shoots per plant and the plant density [*PD*; plants m^−2^] we could calculate the *LAI* [m^2^ leaves on upright shoots m^−2^ ground]:

$$\begin{array}{c} LAI=N_{s}\cdot PD\sum_{k=1}^{N_{l}}LA_{k} \end{array}$$ (2)

where *N*_*s*_ is the number of shoots per plant (two for this study) and *N*_*l*_ is the number of leaves on a shoot.

In order to estimate an *LAI* value for every time point in the experiment, *LAI*(*t*), a logistic growth curve was fitted through *LAI* (from Eq (2)) against normalised time in the growing cycle (i.e. the first day of the cycle is 0 and the day of harvest is 1) yielding a model with an *R*^2^ of 0.98:

$$\begin{array}{c} LAI\left(t\right)=\frac{LAI_{max}}{1+e^{-k(t-t_{0.5})}} \end{array}$$ (3)

where the subscript *max* refers to the maximum *LAI*, *t* is the normalised time in the growth cycle, *t*_0.5_ is the relative time at which half of the *LAI*_*max*_ is reached, and *k* determines the steepness of the curve.

### Disentangling temperature and water vapour effect on *RH* profile

The *RH* results from the combined effect of actual water vapour pressure and temperature (which determines the saturated water vapour pressure). Both can change in the canopy and may have different distribution patterns. There is less radiation lower in the canopy, resulting in a lower temperature and thus higher *RH*. On the other hand, water vapour is added to the canopy by transpiration and humidification of the greenhouse air or removed from the canopy air by advection. The contribution of both temperature and addition/removal of water vapour to vertical differences in *RH* can be determined through factorization. The ratio between *RH* in the canopy, *RH*_*i*_, and ambient *RH*, *RH*_*amb*_, can be written as:

$$\begin{array}{c} \frac{RH_{i}}{RH_{amb}}=\frac{e_{i}/e_{sat,i}}{e_{amb}/e_{sat,amb}} \end{array}$$ (4)

where, *e*, and *e*_*sat*_ are water vapour pressure and saturated water vapour pressure [Pa], respectively. Furthermore, the subscript *amb* refers to the ambient climate and *i* refers to the location of the sensor in the canopy. Following the factorization approach of Poorter et al [18], we can rewrite Eq (4) to:

$$\begin{array}{c} \frac{\text{ln}\left(e_{i}\right)-\text{ln}\left(e_{amb}\right)}{\text{ln}\left(RH_{i}\right)-\text{ln}\left(RH_{amb}\right)}+\frac{\text{ln}\left(e_{sat,amb}\right)-\text{ln}\left(e_{sat,i}\right)}{\text{ln}\left(RH_{i}\right)-\text{ln}\left(RH_{amb}\right)}=1 \end{array}$$ (5)

where the first term gives the fraction of the difference between canopy and ambient *RH* that is associated to a difference in water vapour pressure and thus to the addition or removal of water vapour from the canopy air. The second term gives the fraction of difference between the ambient and the canopy *RH* that is associated to a difference in temperature.

### Statistical analysis

Canopy climate is determined by many interacting processes, especially in a greenhouse where climate can be controlled by a number of climate measures. Simulating the canopy climate would need to take all these processes into account which would complicate the modelling process. Simplifying the model would require insight which processes are important. Here, we quantified the relative importance of the interacting factors that affect canopy climate by using a statistical modelling technique called path analysis [19]. Path analysis aims to analyse the multivariate dependency of variables by specifying causal relationships between variables, and to test whether the posited causal structure is consistent with the data. This allows to quantify direct effects of one variable on another, but also indirect effects of one variable on another, but mediated through a third variable. Unlike dynamic modelling, path analysis does not require to explicitly include or calculate all underlying processes. With this method we worked with the simplest framework that still captures all relevant external factors. Path analysis does not include feedback loops, e.g. from window opening (which is based on ambient temperature) to ambient temperature (which is among others determined by window opening) and vice versa. Therefore, it can be regarded as an intermediate step between standard regression analysis and dynamic modelling. This technique can provide information on the most important processes in the climate system in the greenhouse, without the need to involve the complexity of dynamic modelling.

As a proxy for the difference between ambient to canopy climate, we used the difference between the top sensor above the canopy and the sensor halfway in the canopy (Fig 1), such that a positive difference means that a variable such as temperature is higher in the canopy compared to the ambient climate, and vice versa. The variables that were hypothesised to affect the difference in ambient and canopy air temperature and humidity were: radiation, *LAI* and greenhouse climate measures including supplementary light, temperature of the heating pipe, shading screen closure and window opening. Including these variables in the path analysis implicitly includes relevant processes like transpiration (e.g. in the relation between *LAI* and *q*). Since the growing medium was packed in plastic, soil evaporation, irrigation and soil moisture could be neglected. Note that, in the path analysis, we used absolute humidity instead of *RH*, because *RH* is strongly dependent on temperature; including *RH* would put to much emphasis on this relation. In the path analysis, we defined causal effects and correlations. Causal effects mean that the change in a variable leads to a change in another. In correlations, variables are associated, but no causal direction is specified, either because 1.) there is a feedback between the variables, or 2.) the variables are correlated by variables that were not included in the model (e.g. the climate settings in the greenhouse). For example, all else being equal, there may be a correlation between switching on the heating pipes and the high-pressure sodium (HPS) lamps but one does not causally affect the other. Where possible a causal relationship between the climate control measures and the climate variables (i.e. ambient temperature and humidity, and the difference between ambient and canopy climate) was assumed. However, since there is a strong direct feedback between window opening and ambient temperature, which cannot be incorporated in the path analysis, a correlation was used instead of a causal relation.

As some of the variables were not normally distributed and variance was not heterogeneous across the predictor variables, we applied a generic framework to test multivariate causality based on the notion of d-separation [20]. Based on our knowledge of the system and the degree of model fit, we constructed and modified the causal structure such that the model structure was consistent with the data. The degree of fit of each model was assessed through the Fisher’s C statistic, which follows a chi-square distribution, with 2*k* degrees of freedom, where *k* is the number of missing paths in the model structure [20]. A model was accepted when the p-values derived from Fisher’s C were higher than 0.05, which implies that the causal structure is consistent with the data. Path coefficients were estimated using linear regression or generalised linear models. To account for heteroscedasticity, we selected variance structures best capturing the difference in variance between the variables [21]. Standardised path coefficients were used to assess the relative importance of variables on the variable of interest. Standardised path coefficients measure the effect of one variable on another in standard deviation units. The coefficients were used to compare the importance of direct and indirect effects on the differences between ambient and canopy climate. The indirect effects are calculated by multiplying all the standardised path coefficients on the path from one variable to the variable of interest and by summing the different paths that connect those variables.

A similar path analysis was set up to quantify the relative importance of interacting factors that affect the difference between leaf temperature and canopy air temperature. In this causal model the difference between leaf and canopy air temperature was related to the climate measures temperature of the heating pipe, supplementary lights on/off and opening/closing of the screens and windows. In the analysis, these climate measures were driven by radiation outside the greenhouse.

The hourly-averaged data used as input for the path analysis was a selection of 16 representative days such that different radiation regimes, namely cloudy, partly cloudy and sunny, and differences in *LAI* during a growing cycle were equally represented in the dataset (S2 Table). Note that, for inside and outside radiation measurements, global radiation (outside: 250-3000 nm; inside: 300-2800 nm) is used and not *PAR* as used for the comparison between different weather types. For the path analysis on leaf temperature, the dataset was further limited to days where the leaf temperature was measured. Leaf temperature measurements of two leaves at the same height were averaged to reduce the effect of differences between leaf temperature and canopy climate, because leaf temperature was not measured at exactly the same location as the canopy climate. Data for the path analysis was averaged to hourly values and included both day and night time measurements. The data were analysed in R 3.5.0 [22].

## Results

### Ambient and canopy climate differ greatly

**Fig 2:**
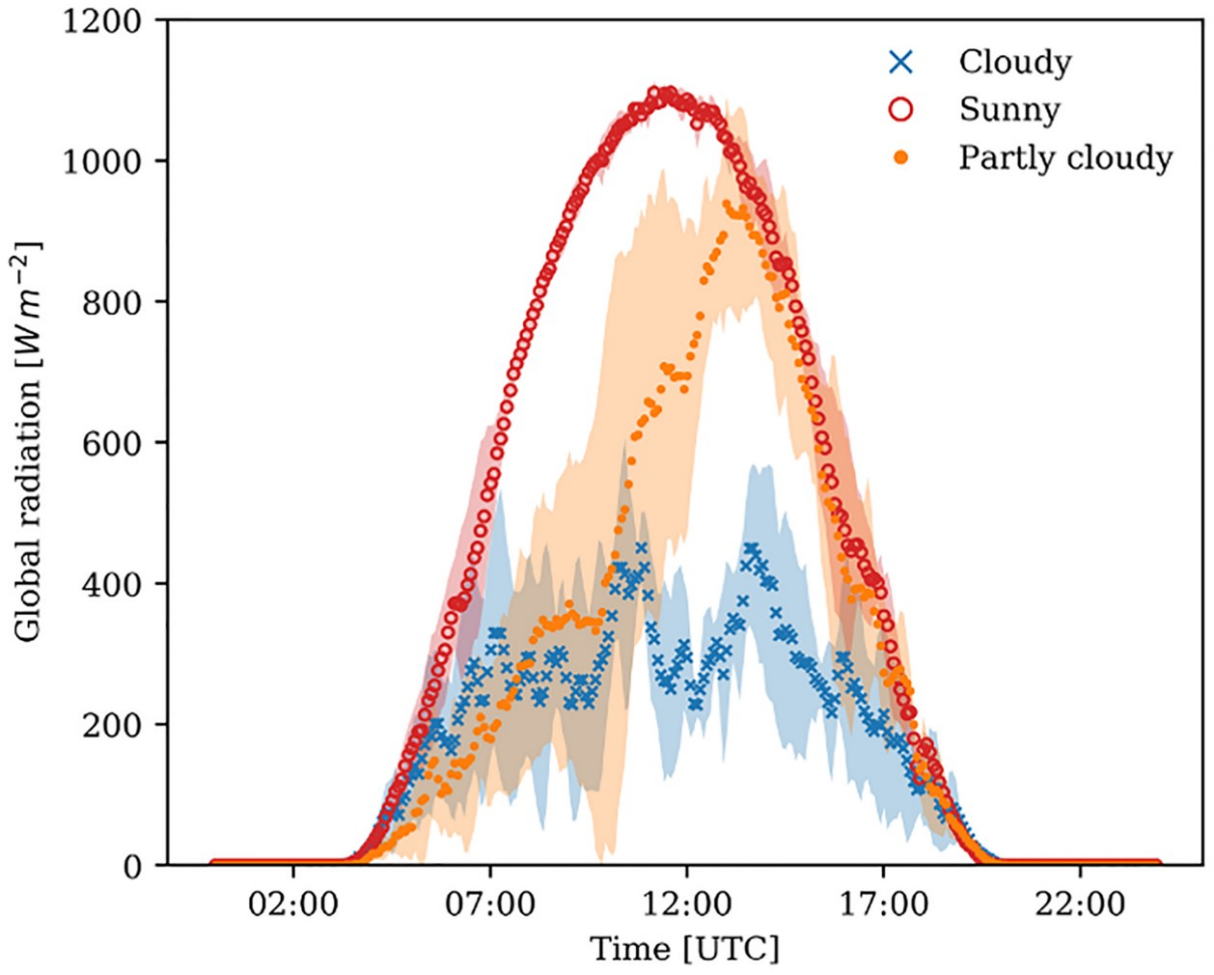
Global radiation outside the greenhouse. Data averaged for 5 sunny (red), 4 cloudy (blue), and 4 partly cloudy days (orange). The dotted lines represent the averages, and the shaded area the standard deviation of the specific weather types.

The largest variation in terms of daily incoming radiation was caused by clouds. Since, global radiation was measured outside the greenhouse, fluctuations are solely due to clouds. The sunny days showed a clear bell-shaped curve (Fig 2). Since the selected days were close to the summer solstice, they represented almost the highest radiation possible on sunny days.

**Fig 3:**
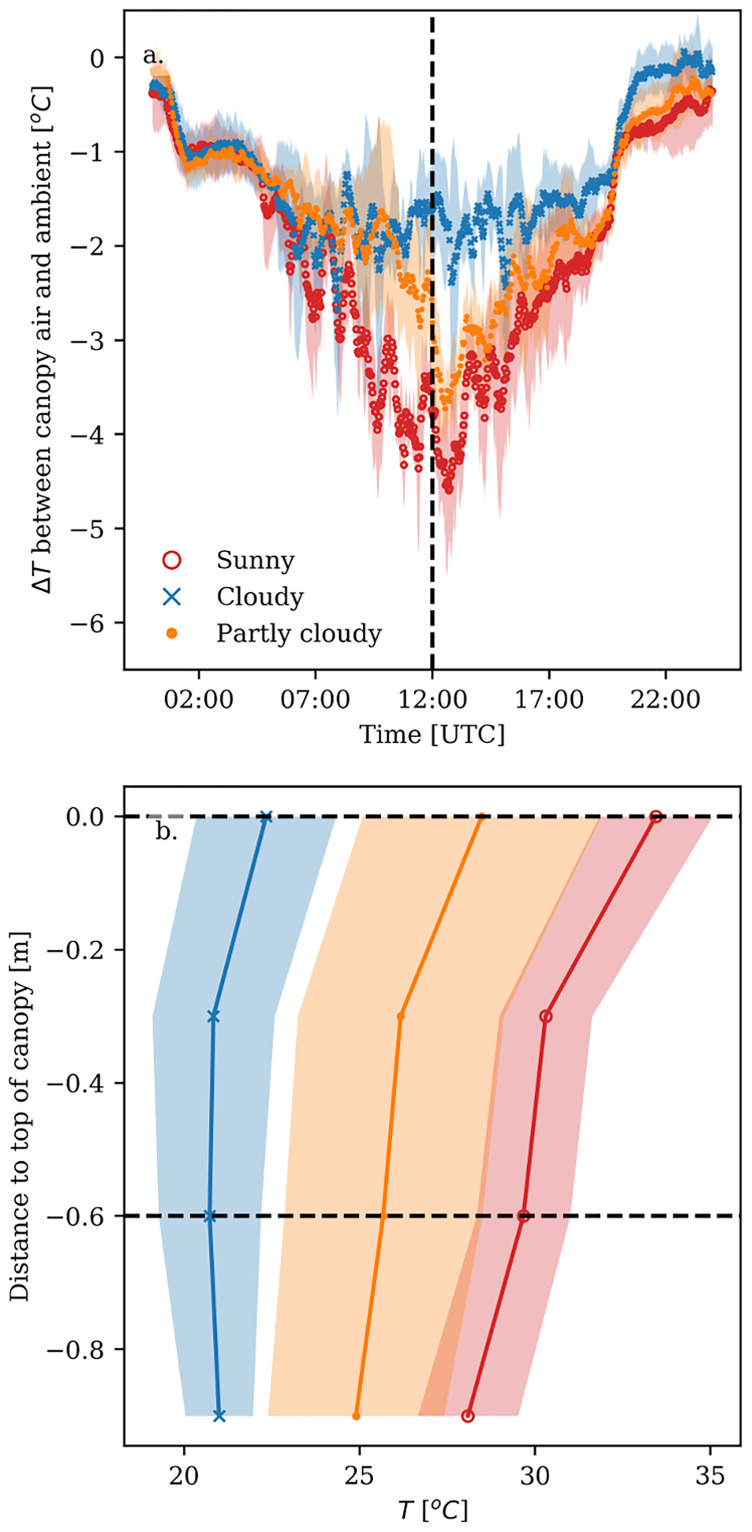
Difference between ambient temperature and canopy air temperature (i.e. air temperature 60 cm below the top of the canopy). Bold lines represent the average value, whereas the shaded area represents the standard deviation over the days for sunny (red), cloudy (blue) and partly cloudy days (orange). (a) time course of the temperature difference, where the dashed line indicates noon. (b) vertical temperature profile in the canopy at noon; the dashed lines show the heights at which the differences between ambient and canopy air temperature were calculated.

The differences between ambient and canopy air temperature were largest around noon, when the radiation was highest, and smallest at dawn and dusk. Under sunny conditions, the canopy air temperature was up to 5°C lower than the ambient temperature at noon (Fig 3). In contrast, on the cloudy days the canopy air temperature was at most 2°C lower than the ambient temperature around noon. The difference between ambient and canopy air temperature on the partly cloudy days followed the pattern of the cloudy days before noon. After noon the difference resembled the pattern of the sunny days, because there were more clouds before noon than after noon. The canopy air temperature also decreased from the top towards the bottom in the canopy (Fig 3). However, when the heating pipe was used to heat the greenhouse, the canopy air temperature increased lower in the canopy.

**Fig 4:**
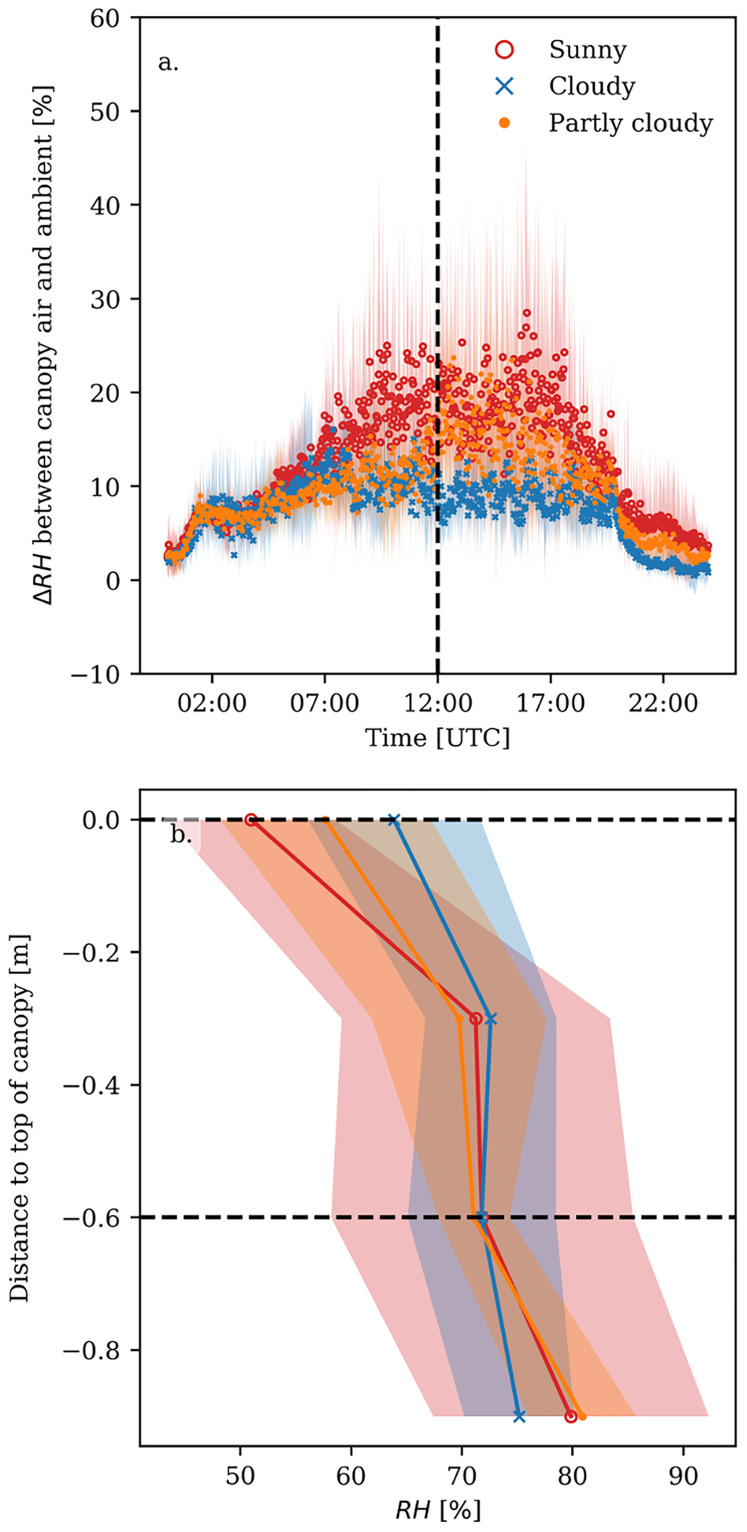
Difference between ambient relative humidity (*RH*) and canopy relative humidity (i.e. *RH* of the air 60 cm below the top of the canopy). Bold lines represent the average value, whereas the shaded area represents the standard deviation over the days for sunny (red), cloudy (blue) and partly cloudy days (orange). (a) time course of the *RH* difference, where the dashed line indicates noon. (b) vertical *RH* profile in the canopy at noon; the dashed lines show the heights at which the differences between ambient and canopy *RH* were calculated.

While the temperature decreased from top to bottom of the canopy, the *RH* increased. The differences between ambient and canopy *RH* were substantially larger on sunny than on cloudy days (Fig 4). For the sunny days, the *RH* was about 25% higher in the canopy than in the ambient air, with peaks up to 40% during humidification events in the greenhouse. The difference between ambient and canopy *RH* was about 15% on the cloudy days. Lower in the canopy the *RH* difference was even larger.

**Fig 5:**
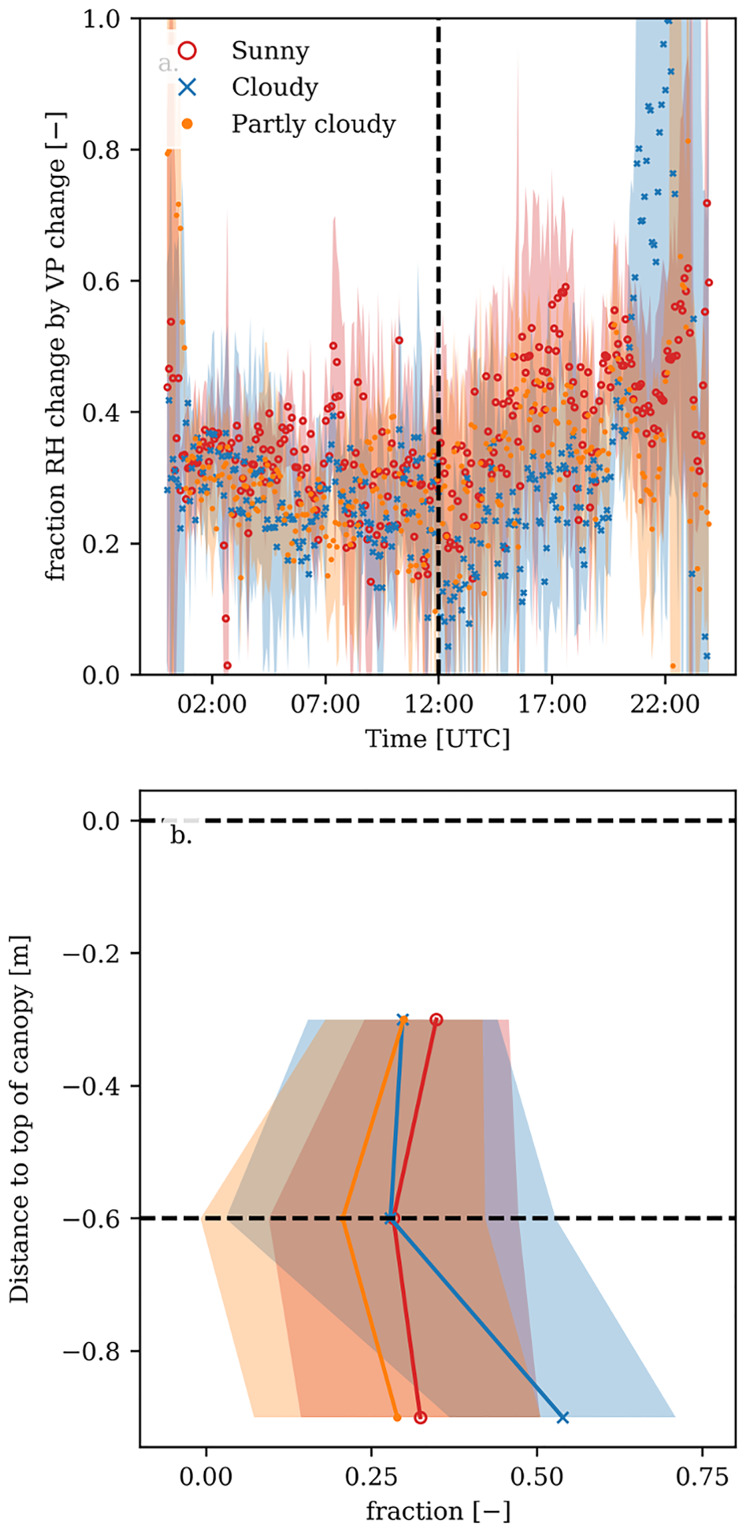
Fraction of the difference between ambient relative humidity (*RH*) and canopy relative humidity (i.e. *RH* of the air 60 cm below the top of the canopy) that is determined by the change of water vapour pressure. Fraction of the *RH* difference determined by changes in water vapour pressure (bold lines) for sunny (red), cloudy (blue) and partly cloudy days (orange). The shaded area represents the standard deviation. (a) time course of the fraction, where the dashed line indicates noon. (b) vertical profile of the fraction in the canopy at noon; the dashed lines show the heights at which the fraction between ambient and canopy *RH* explained by the change of water vapour pressure were calculated.

For all types of days the day-time difference in *RH* was mostly determined by the temperature difference, as calculated based on Eq 4. That is, 60-90% of the observed variation in *RH* with depth in the canopy could be attributed to a reduction in temperature, which directly determines the saturation vapour pressure (Fig 5 and S3 Fig). That means that 10-40% of the observed variation can be attributed to a change in the vapour pressure itself, due to the addition or removal of water vapour. This addition or removal comes from humidifying the greenhouse and transpiration by the plant. Evaporation from the soil can be neglected, since the growing medium was packed in plastic. Interestingly, the relative contribution of the temperature effect and the vapour pressure effect on variations in *RH* changed with canopy depth. Higher in the canopy the *RH* difference was mostly determined by temperature, but the contribution of water vapour pressure increased from top towards lower in the canopy (Fig 5b and S4b Fig).

### Difference between ambient and canopy climate directly affected by radiation and *LAI*

**Fig 6:**
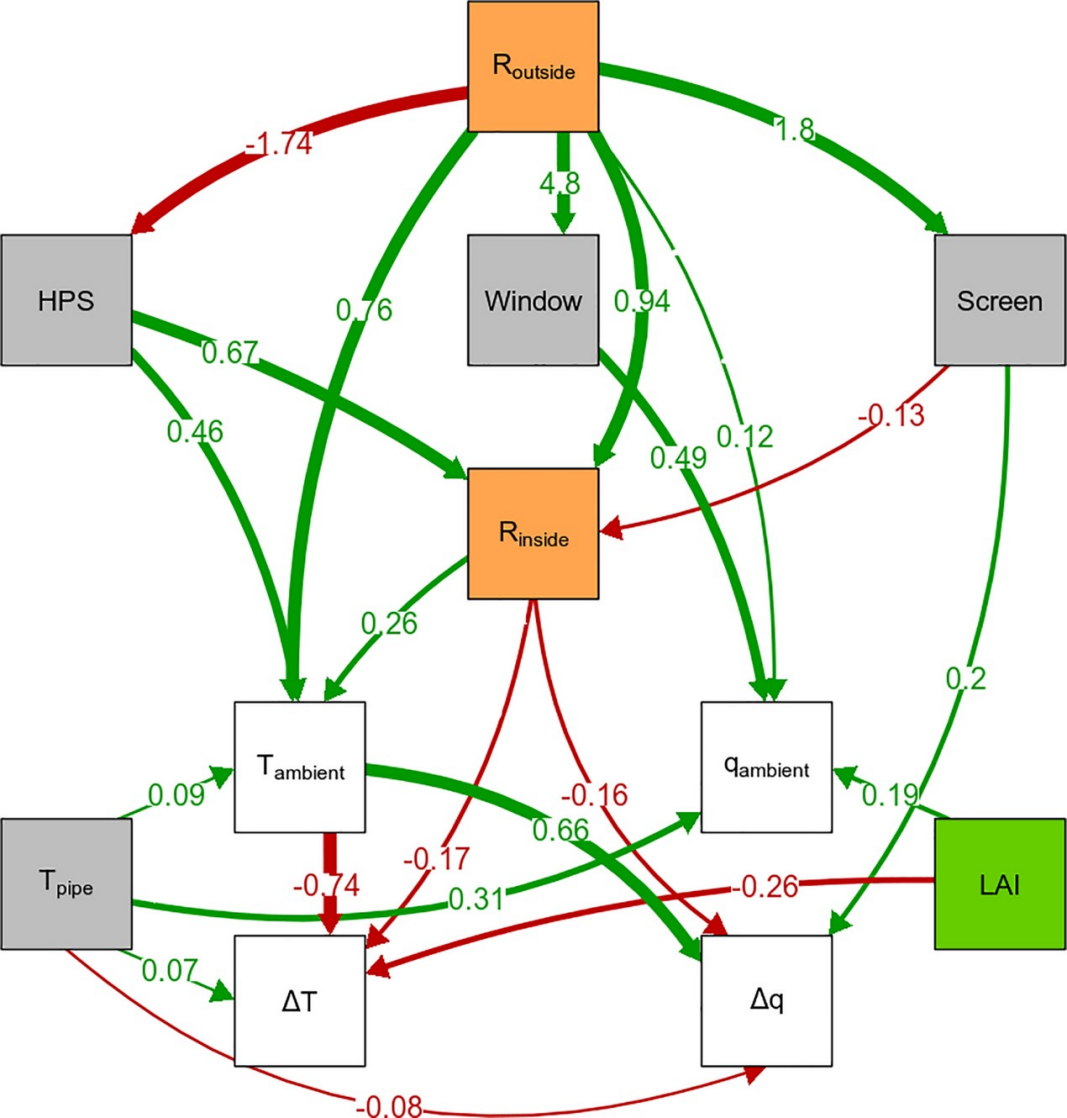
Causal path model for the difference between canopy air and ambient temperature (Δ*T*) and absolute humidity (Δ*q*). Causal path model that was consistent with the data (p = 0.058, df = 32, Fisher’s C = 45.47), where arrows show a causal relation and the values the standardised path coefficients. The standardised path coefficients measure the effect of one variable on another in standard deviation units. The colour of the boxes denote the following: *white* represents the climate variables, *grey* represents the climate control measures, *green* represents the crop and *orange* represents radiation. Paths with a standardised path coefficient below 0.07 have been removed for presentation purposes. For the complete diagram see S5 Fig. *Abbreviations: R_outside_ is outside global radiation; R_inside_ is inside global radiation; HPS is High Pressure Sodium lamps, supplemental assimilation lighting; Window is window opening (open or closed); Screen is shading screen position (open or closed); T_pipe_ is temperature of the heating pipe; LAI is leaf area index; T_ambient_ is ambient temperature, temperature above canopy; q_ambient_ is ambient absolute humidity, absolute humidity above canopy*; Δ*T is difference between ambient temperature and air temperature in the canopy*; Δ*q is difference between ambient absolute humidity and absolute humidity in the canopy. Also see* Table 1.

Based on path analysis (Fig 6 and S5 Fig and S3 Table), we found that the temperature difference between ambient and canopy, Δ*T*, was determined by variables from three categories, namely crop characteristics, ambient conditions and greenhouse climate measures (p = 0.058, df = 32, Fisher’s C = 45.47). Note that Δ*T* was calculated as canopy air temperature minus the ambient temperature, *T*_*canopy*_ − *T*_*ambient*_. Since in our experiment the temperature in the canopy was usually lower than the ambient temperature, a negative coefficient in the path analysis implies that the temperature difference between ambient and canopy becomes larger. An increase in *LAI* increased the temperature difference (path coefficient = -0.26; i.e. the canopy air became relatively cooler). This is logical as a larger *LAI* entails a stronger gradient in radiation capture and in associated convective warming of the air.

**Table 1:** List of definition of all the used abbreviations.

Nomenclature		
Δ*q*	[%]	Difference between canopy air and ambient absolute humidity
Δ*RH*	[%]	Difference between canopy air and ambient relative humidity
Δ*T*_*leaf*_	[°C]	Difference between canopy air and leaf temperature
Δ*T*	[°C]	Difference between canopy air and ambient temperature
*e*_*amb*_	[%]	Saturated vapour pressure at top of canopy
*e*_*amb*_	[%]	Vapour pressure at top of canopy
*e*_*i*_	[%]	Saturated vapour pressure at location *i* in canopy
*e*_*i*_	[%]	Vapour pressure at location *i* in canopy
*HPS*		High pressure sodium supplementary light
*L*	[m]	Length of single leaf
*LA*	[m^−2^]	Leaf area of single leaf
*LAI*_*max*_	[-]	Maximum leaf area
*LAI*	[-]	Leaf Area index
*N*_*l*_	[#]	Number of leaves on a shoot
*N*_*s*_	[#]	Number of shoots per plant
*N*	[#]	Number of leaflets of single leaf
*PAR*	[*μ*mol m^−2^ s^−1^]	Photosynthetically active radiation
*PD*	[# m^−2^]	Plant density
*q*_*ambient*_	[g kg^−1^]	Ambient absolute humidity
*q*_*canopy*_	[g kg^−1^]	Canopy absolute humidity
*R*_*inside*_	[W m^−2^]	Global radiation inside greenhouse
*R*_*outside*_	[W m^−2^]	Global radiation outside greenhouse
*RH*_*amb*_	[%]	Relative humidity at top of canopy
*RH*_*i*_	[%]	Relative humidity at location *i* in canopy
*t*_0.5_	[-]	Normalised time at which half of *LAI*_*max*_ is reached
*T*_*ambient*_	[°C]	Ambient greenhouse temperature
*T*_*canopy*_	[°C]	Canopy temperature
*T*_*leaf*_	[°C]	Leaf temperature
*T*_*pipe*_	[°C]	Heating pipe temperature
*W*	[m]	Width of single leaf

Next to the *LAI*, the radiation level was important for determining the temperature difference. More radiation in the greenhouse (*R*_*inside*_) increased the temperature difference (path coefficient = -0.17). A high radiation above the canopy entails a larger absolute difference in radiation absorption between the top and bottom of the canopy, which likely results in a larger difference in leaf temperatures and thus in a larger difference between ambient and canopy air temperature. The ambient temperature (*T*_*ambient*_) itself increased the difference between ambient and canopy temperature (path coefficient = -0.74). This is inherent to the way we calculated the difference. When *T*_*canopy*_, which was most often cooler than *T*_*ambient*_, stays the same and *T*_*ambient*_ increases, the temperature difference becomes larger.

With the climate control settings used in our greenhouse, one of the greenhouse climate control measures that directly influenced the temperature difference (indirect effects of climate measures will be discussed in the next section) was the temperature of the heating pipe (*T*_*pipe*_). A high temperature of the heating pipes below the canopy decreased the temperature difference (path coefficient = 0.07; i.e. the canopy air became relatively warmer) because they mostly influenced the lower part of the canopy. Another climate control measure that directly influenced the temperature difference was the use of HPS lamps. The HPS lamps directly influenced the temperature difference in two ways. First, using HPS lamps increased the shortwave radiation (via *R*_*inside*_; coefficient = -0.11; Fig 6). Second, there was a small direct effect due to the use of HPS lamps (i.e. not by changing shortwave radiation, *R*_*inside*_) that decreased the temperature difference (path coefficient = 0.02; S5 Fig). Due to their positioning, the emitted light from HPS lamps comes from many directions and high elevation angles. As such the vertical light distribution was more homogeneous and the top leaves were cooler compared to only direct light, because of less light absorption in the top [23, 24].

The absolute humidity difference in a cut-rose canopy, Δ*q*, was mainly influenced by variables from two categories related to ambient conditions and greenhouse climate measures. A higher *LAI* only slightly increased the humidity difference (path coefficient = 0.06; i.e. the canopy air became relatively more humid). Radiation decreased the humidity difference, because higher radiation increased the difference in absolute radiation absorption, which likely resulted in a similar difference in leaf temperature and thus transpiration. The radiation inside the greenhouse decreased the absolute humidity difference (path coefficient = -0.16). A higher ambient temperature with the same canopy air temperature can create more stable conditions. More moisture is trapped in the canopy with higher stability, because there is less exchange between the canopy and the ambient air, hence the ambient temperature increased the absolute humidity difference (path coefficient = 0.66).

The heating pipes underneath the canopy (*T*_*pipe*_) and the shading screen were the greenhouse climate measures that influenced the humidity difference. By heating the bottom part of the cut-rose canopy, the heating pipes induced mixing. Therefore, the heating pipes reduced the absolute humidity difference (path coefficient = -0.08). The shading screen reduced ventilation [25] and thus mixing inside the greenhouse, hence the humidity difference was increased (path coefficient = 0.2).

There were strong correlations between ambient temperature and window opening (correlation coefficient = 0.32), and between ambient temperature and humidity (correlation coefficient = 0.61). Also, the difference between ambient and canopy temperature and humidity showed a relatively strong correlation (correlation coefficient = -0.35). Additionally, there are many correlations between the climate control measures, such as supplemental lighting, shading screens, window opening and the temperature of heating pipes. These correlations show that the climate control measures are all based on a set of climate control rules and that they affect the climate all at the same time.

### Climate differences can be influenced by management practices

Outside radiation is an external condition that cannot be controlled. However, in the greenhouse, climate control measures can be used to modify the difference between ambient and canopy climate (Fig 6 and Table 2). In this study, supplementary lighting had the largest effect on the difference between canopy and ambient temperature and humidity, where turning on the lights increased the temperature and humidity difference with 0.33°C (note that negative values in Table 2 for Δ*T* mean a larger negative difference) and 0.16 g kg^−1^. The effect of the shading screens and the temperature of the heating pipes on the difference between canopy and ambient climate was small. The effect of window opening could not be studied, since there was a strong direct feedback between ambient temperature and window opening (i.e. window opening was determined by the ambient temperature). Considering the window opening was a binomial value with either fully open or closed, the correlation between the window opening and ambient temperature was high (correlation coefficient = 0.32; S3 Table).

**Table 2:** Absolute direct and indirect effects of climate control measures on the difference between ambient and canopy air temperature, Δ*T*, and absolute humidity, Δ*q*, and on the difference between canopy air and leaf temperature, Δ*T*_*leaf*_. Values are obtained by multiplying the coefficients in a path and adding the multiplied coefficients from different paths. Note that negative values for Δ*T* mean a larger negative difference.

Variable		HPS	*T*_*pipe*_	Screen	Window^a^
Δ*T*	direct	0.04	0.01	-	-
	indirect	-0.37	-0.02	0.04	-
Δ*q*	direct	-	-0.01	0.10	-
	indirect	0.16	0.01	0.00	-
Δ*T*_*leaf*_	direct	0.13	-0.06	-	-
	indirect	-0.08	-	0.17	-

^a^ The effect of window could not be calculated, since there was a strong direct feedback between ambient temperature and window opening. Window opening is added in the table for completeness.

### Difference between canopy air and leaf temperature mostly affected by radiation

**Fig 7:**
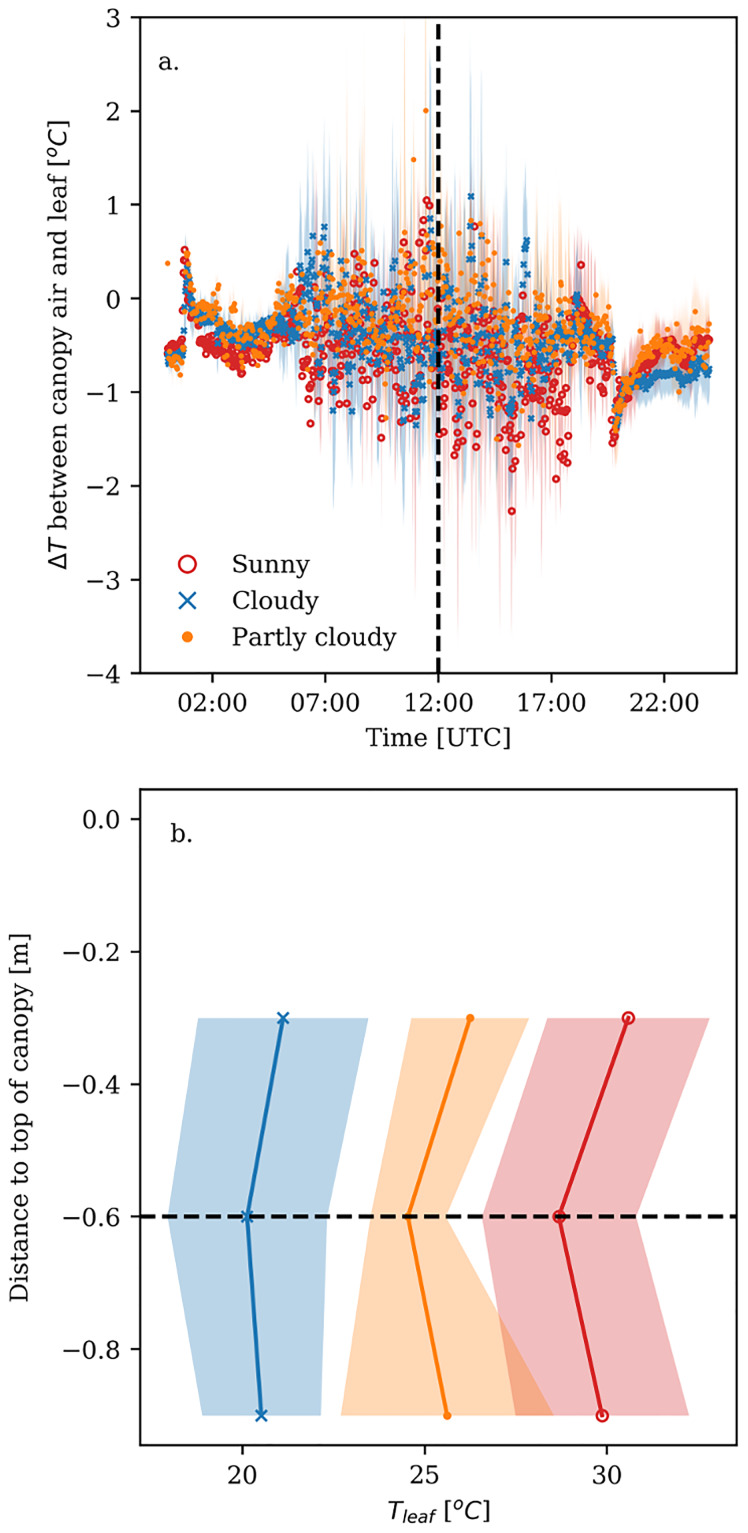
Difference between canopy air and leaf temperature both 60cm below the top of the canopy. Bold lines represent the average value, whereas the shaded area represents the standard deviation over the days for sunny (red), cloudy (blue) and partly cloudy days (orange). a.) time course of the temperature difference, where the dashed line indicates noon. b.) vertical leaf temperature profile at noon; the dashed line shows the height at which the difference between canopy air and leaf temperature was calculated.

During daytime (3:30—19:40 UTC) the difference between canopy air and leaf temperature fluctuated much more compared to nighttime, around an average close to zero (Fig 7). Overall, the difference between canopy air and leaf temperature was relatively similar for the different types of days. Combined with the difference between canopy air and ambient temperature, the differences between ambient and leaf temperature ranged from +1 to -7°C (S7 Fig).

**Fig 8:**
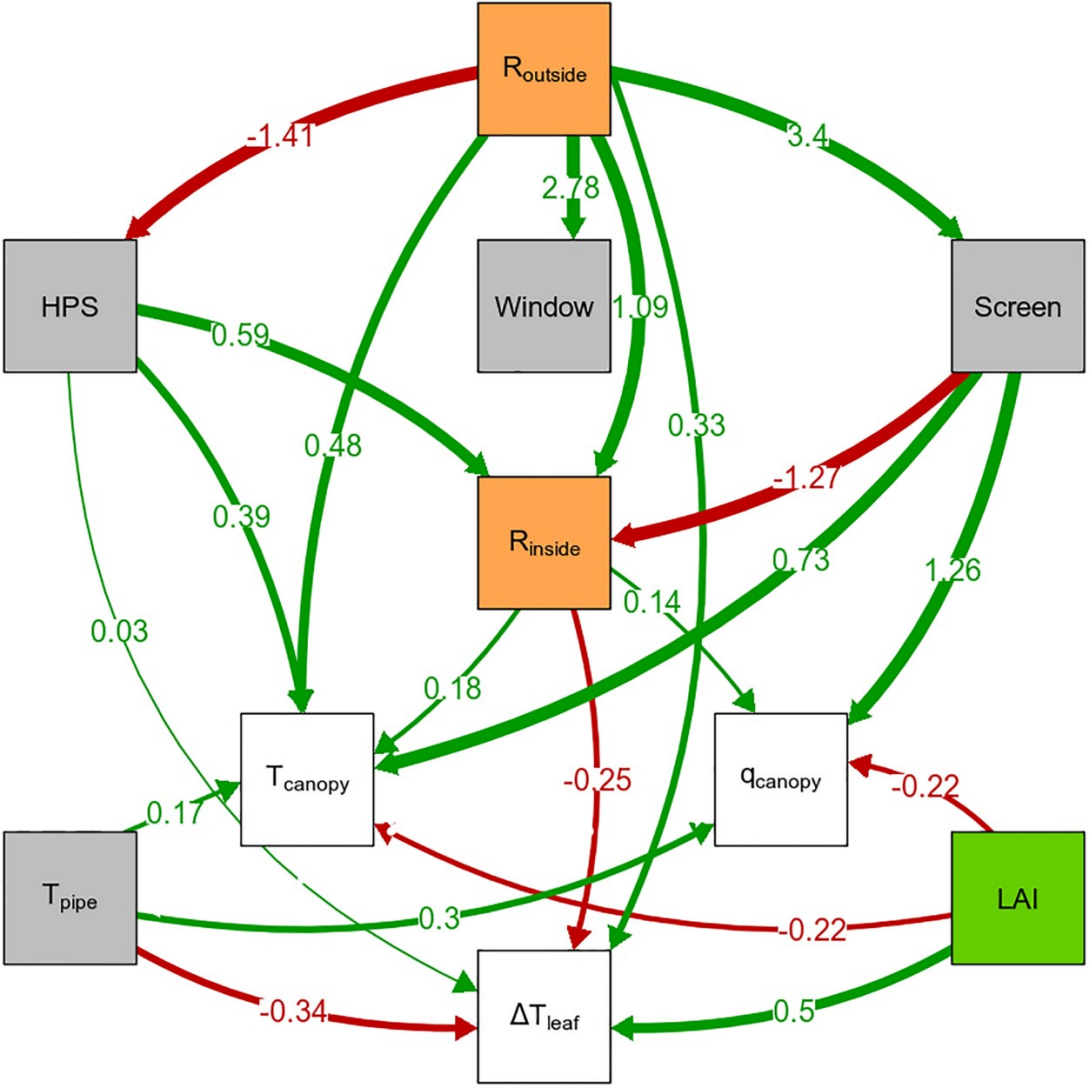
Causal path model for the difference between canopy air and leaf temperature. Causal path model that was consistent with the data (p = 0.062, df = 32, Fisher’s C = 45.15), where arrows show a causal relation of standardised path coefficients (values at arrows). The colour of the boxes denote the following: *white* represents the climate variables, *grey* represents the climate control measures, *green* represents the crop and *orange* represents radiation. For the complete diagram see S8 Fig. *Abbreviations: R_outside_ is outside global radiation; R_inside_ is inside global radiation; HPS is High Pressure Sodium lamps, supplemental assimilation lighting; Window is window opening (open or closed); Screen is shading screen position (open or closed); T_pipe_ is temperature of the heating pipe; LAI is leaf area index; T_ambient_ is ambient temperature, temperature above canopy; q_ambient_ is ambient absolute humidity, absolute humidity above canopy*; Δ*T_leaf_ is difference between canopy air temperature and leaf temperature. Also see* Table 1.

The components of the energy balance: net radiation, and sensible and latent heat flux are usually strongly influenced by temperature, humidity and radiation. Based on the path analysis (Fig 8 and S4 Table), we found that, in our experiment, the temperature difference between canopy air and leaf temperature was determined by radiation, *LAI*, temperature of the heating pipe and the use of supplementary light (p = 0.062, df = 32, Fisher’s C = 45.15). Interestingly, all the variables are strongly related to radiation, because the temperature of the heating pipe represents the thermal (i.e. infra-red) radiation and the *LAI* determines the amount of light intercepted above the leaves of interest. Note that the difference between canopy air and leaf temperature was calculated as canopy air temperature minus leaf temperature, *T*_*canopy*_ − *T*_*leaf*_. Since the leaf temperature of the cut-roses was usually lower than the canopy air temperature, a positive coefficient implies that the temperature difference between canopy air and leaf temperature became larger.

The *LAI* increased the difference between canopy air and leaf temperature (path coefficient = 0.50). Contrary, a higher temperature of the heating pipe emits a lot of thermal (i.e. infra-red) radiation that heats up the leaf, thereby decreasing the temperature difference between canopy air and the leaf (path coefficient = -0.34). The direct effect of radiation (i.e. the combined effect of radiation outside and inside the cut-rose greenhouse) was small, because the coefficients of radiation outside and inside the greenhouse had a similar, but opposite value (path coefficient = 0.33 and -0.25).

The temperature difference between canopy air and the leaf could be influenced by using the heating pipes and the shading screens. Increasing the temperature of the heating pipe with 1°C decreased the canopy air and leaf temperature difference with 0.06°C (Table 2). When the leaf was warmer than the canopy air, the screens reduced the temperature difference with 0.17°C.

Interestingly, in our greenhouse setting, only the radiation component of the leaf energy balance turned out to be significant in the path model. This showed that canopy air temperature and absolute humidity did not significantly affect the difference between leaf and canopy air temperature.

## Discussion

### Consequences of large difference between ambient and canopy climate on plant and disease development

By measuring the ambient and canopy climate at different vertical locations in the greenhouse, we showed that canopy air temperatures could be up to 5°C lower than the ambient temperature and that there was a strong variation in this difference over time. In general, we found that temperature decreased from top towards the bottom of the canopy. Leaves in the top of the canopy receive most of the radiation and will thus be warmer than leaves lower in the canopy. Consequently, the air close to the leaves becomes warmer in the top of the canopy than lower in the canopy. Differences in ambient and canopy air temperature in this study are in agreement with the differences ranging from -2 to -9°C found in greenhouses [26, 27]. Overall the difference between canopy to ambient temperature depended mostly on overall climate conditions, i.e. the amount of radiation and ambient temperature, climate control measures in the greenhouse (e.g. supplementary lighting and screening) and canopy characteristics (i.e. *LAI*). Clouds not only decrease the incoming radiation, but also increase the fraction of radiation that is diffuse [28]. Therefore, clouds decrease the difference between ambient and canopy air temperature for two reasons: first, because there is less radiation to heat the top leaves; and second because light that is more diffuse tends to penetrate deeper into the canopy [23], resulting in more radiation on the lower leaves. This allows lower leaves to become relatively warmer compared to the top leaves. This temperature difference between the canopy air and the ambient air can be influenced by climate control measures (see the last section of the Discussion). We showed that the leaf temperature fluctuated enormously between 2^*o*^ *C* higher and lower than the canopy air temperature, which is of the same range as the difference between air temperature and apex temperature in tomato [2]. Combining this with the difference between canopy air and ambient temperature, the difference between ambient and leaf temperature ranged from +1 to -7°C.

During the dark period, the difference between ambient and canopy air temperature were very small and sometimes even reversed, compared to the light period. During this dark period, thermal (i.e. infra-red) radiation is crucial for the energy balance of the canopy and the leaves. Top leaves loose more radiation to the cooler greenhouse cover or screens than lower leaves, thereby, diminishing the difference between ambient and canopy air temperature. This is greatly influenced by the presence of clouds outside, which increase the greenhouse cover temperature, and the use of screens, because both will reduce the losses of radiation by the top canopy and reduce cooling at the top.

The canopy *RH* was on average 25% (with peaks to 40%) higher than the *RH* outside the canopy around noon, which is similar to *RH* ranges found by previous studies [5, 29]. Most of the variation in the *RH* profile was due to the temperature profile, i.e. lower canopy air temperatures leading to lower saturated vapour pressures and concomitantly higher *RH* values. The change of water vapour pressure explained 20 to 40% of the variation in the *RH* profile (Fig 5).

Many experiments entail comparisons between plants that vary in traits such as growth rate, leaf size, specific leaf area and biomass allocation to leaves, or involve treatments such as plant density which in turn may strongly affect *LAI*. Although many of these experiments were done in greenhouses with a controlled enviro nment, we showed that one m^2^ leaves per m^−2^ ground area increased the difference between ambient and canopy temperature with 0.15°C. It is important to take this effect of *LAI* into account, as it is the organ temperatures and not ambient temperatures that determine key physiological processes usually of interest to researchers such as photosynthesis, respiration and organ development rates, e.g. leaf expansion rate [30] or leaf initiation rate [31]. Furthermore, the plant phenotype is determined by the canopy climate and vertical differences therein. For example, a different apical bud temperature compared to leaf temperature, which is closely related to canopy air temperature, can result in different phenotypes [32]. For instance, a 3°C warmer apical bud compared to (mean) leaf temperature decreases the final leaf area with approximately 25% in cucumber (*Cucumis sativus*), where the temperature difference effect increased with height [32].

Furthermore, when considering developmental effects for a given developmental process (e.g. flower initiation), temperatures were 15—30°C above the base temperature of approximately 5°C [33]. Assuming a linear relation between developmental rate and temperature, organ temperatures of 3°C below the ambient temperature (commonly found in this study) would entail a 10—20% lower developmental rate than based on ambient temperature. Similar reasoning applies for pest and disease development. Additionally, a 10-20% higher *RH* in the canopy can greatly increase the chance of leaf wetness [34] of which the duration required by certain pathogens to develop changes with temperature ([e.g. minimum leaf wetness period required for germination at 25°C is 12 hours and goes to 9 hours at 20°C; [35]).

Finally, accurate assessment of canopy climate and leaf temperature is not only essential for the interpretation of experiments, but also in crop yield predictions using models. Crop simulation models are widely used to e.g. predict yields as a function of enviro nmental conditions, including the effects of climate change. These models are used for field and greenhouse crops [36–38]. Crop growth models, of both, often assume a vertically homogeneous climate. This applies for field crop models, such as LINTUL [39–41], GECROS [42], DSSAT [43] or greenhouse models (e.g. [38, 44, 45]). It is especially important to consider the vertical differences for non-linear processes, like photosynthesis. For instance, not taking into account vertical temperature differences may result in 20-30% underestimation of leaf respiration lower in the canopy [16] and overestimate yield predictions [46]. Therefore, crop models should account for differences between climate above the canopy and within the canopy.

### Consequences of large difference between ambient and canopy climate on modelling plant-climate system in greenhouses

Especially in greenhouses, canopy climate results from a complex interplay of a wide range of factors. The path analysis approach used in this study allowed us to get insight in the factors that determine the canopy climate. The analysis showed that the difference between ambient and canopy temperature was determined by the *LAI*, the ambient temperature, radiation (and all the climate control measures that influence radiation) and temperature of the heating pipes below the canopy. For the humidity difference the same applied with only the addition of the shading screens.

In the path analysis approach, it is not possible to include feedback loops. However, feedbacks are present in the greenhouse-canopy system, as for example, ambient temperature affects canopy air temperature, which drives leaf growth which in turn feeds back to the canopy and ambient climate. In the path analysis feedback loops were represented by correlations. Important correlations in the path model that represented feedback loops were the relation between ambient temperature and window opening; between ambient temperature and humidity and also the correlation of the difference between ambient and canopy temperature with the difference between ambient and canopy humidity. Especially, the correlation between ambient temperature and humidity, was very strong. This shows that a higher humidity resulted in more closed stomata and thus less transpirational cooling, or that temperature increased humidity by increasing transpiration. The same reasoning applies to the correlation of the difference between ambient and canopy temperature with the difference between ambient and canopy humidity.

Many of the different greenhouse climate measures correlated with each other, such as supplemental lighting, shading screens, window opening and the temperature of heating pipes. They correlated, because all these measures were mostly determined by the outside global radiation and ambient temperature. The climate measures that were mostly used in cooler, low-light conditions were positively correlated with each other, just like the climate measures that were used in warm, high-light conditions. Correlations between these groups were negative, except for the correlation between heating pipe temperature and screens. This might be, because both were also used during the night, to heat the greenhouse and prevent radiative cooling.

Many causal relations and correlations related to the climate measures were found the be the same for both the path analysis of the difference between ambient and canopy climate, and the path analysis of the difference between leaf and canopy air climate, e.g. the relation between supplementary lighting and inside global radiation. Especially the factors describing the different climates, such as ambient and canopy air temperature and absolute humidity, and differences between the different climates (variables denoted with a Δ), were observed to be different. This has to do with the fact that the values of the path and correlation coefficients depend on the whole causal path model. Since the factors describing the different climates are different for the two causal path models, the largest differences between the path and correlation coefficients of these two causal path models were related to these factors.

For a detailed analysis on the dynamic interaction between plant and greenhouse climate, dynamic simulation models are required. Furthermore, plant characteristics like leaf area and architecture determine the climate differences in the canopy. Thus, to study how other plant traits influence the climate gradients, and how this feeds back on plant functioning, a functional-structural plant (FSP) model that can simulate canopy architecture in 3D over time [47] needs to be linked to a canopy climate model (e.g. [48]). Such a model would also be instrumental in studying horizontally heterogeneous canopies such as rose. A combined FSP-canopy climate model could be used to quantify the extent to which different processes determine the climate gradients in a greenhouse. While an FSP model can mechanistically capture the plant-climate interactions, implementing the effect of external factors on those plant-climate interactions, such as the effect of window opening on the difference between canopy air and ambient temperature, is not straightforward. The effect of these external factors could be captured by a combination of such an FSP model with the relations and coefficients of the path analysis presented here. For instance, the effect of window opening on the difference between canopy air and ambient temperature could be accounted for by adding the effect of window opening on the difference between canopy air and ambient temperature, as obtained from the path analysis (Table 2).

The path analysis on the difference between leaf and canopy air temperature revealed that the radiation component was the most important in determining the difference, since a higher *LAI* usually results in higher radiation absorption. Net radiation is very important, because it determines the amount of available energy to divide over the different components of the energy balance, namely sensible and latent heat flux and heat storage [49]. Heat storage, that determines the difference between canopy air and leaf temperature, is the closing term of the energy balance. The energy not used for sensible and latent heat flux is stored in the leaf, as such one would expect that the latent and sensible heat flux should also influence the temperature difference. Our analysis shows that the effect of latent and sensible heat on the difference between leaf and canopy air temperature is mostly regulated by the amount of available radiation, since canopy air temperature and humidity did not affect the difference. This shows that for the development of a combined FSP-canopy climate-leaf temperature model, it is very important to have a correct representation of the radiation components of the energy balance, i.e. both shortwave and longwave radiation.

### How could climate differences be controlled?

The outside radiation coming from the sun cannot be controlled, but does strongly influence the ambient and canopy climate, and leaf temperatures. In the greenhouse, the amount of radiation can to some extent be controlled, e.g. by opening/closing the shading screens or turning on/off the supplementary light. However, we showed that these climate measures influenced not only the incoming radiation, but also the difference between ambient and canopy climate. We showed that supplementary lighting had the biggest effect on the difference between canopy air and ambient temperature among all analysed climate control measures. Turning off the lights (600W HPS with 150 *μmol m*^−2^ *s*^−1^ at top of canopy) would reduce the temperature difference with 0.38°C. However, this is not enough to make the ambient and canopy climate similar, since differences range from 2-5°C at noon.

The effect of window opening could not be studied, because of the direct feedback between ambient temperature and window opening. However, they could be positively correlated because opening of the windows increases the ventilation area, which can reduce the greenhouse ambient temperature [50]. In turn, reducing ambient temperature reduces the difference between canopy and ambient air. Thus, opening the windows when there is high radiation could help to reduce the temperature difference and have the canopy air temperature closer to ambient temperature.

The temperature of the heating pipes was not very important for the ambient and canopy air temperature difference. However, it decreased the difference between leaf and canopy air temperature (S4 Table). Therefore, the heating pipes could be used to get the leaf temperature closer to the canopy air temperature. This makes it easier to control the leaf temperature, since it is easier to measure the canopy air temperature than the leaf temperature. On the other hand, the heating pipes increase the difference between ambient and canopy air temperature, thus the canopy air temperature is lower than the measured ambient temperature, which increases the risk of dew formation and thus the probability of infection by diseases. However, the effect of the heating pipes on the difference between leaf and canopy air temperature is larger than on the difference between ambient and canopy air temperature.

## Conclusions

In this study, we aimed to quantify the relative importance of the ambient climate, climate control measures and the developing canopy on 1.) the canopy climate, and 2.) the leaf temperature.

We showed that differences between the ambient and canopy climate can be large. Temperature differences ranged from a 2-5°C cooler canopy air temperature compared to the ambient temperature. Furthermore, the relative humidity, *RH*, can be 15-25% higher in the canopy compared to the ambient *RH*. We showed that radiation and leaf area index are very important for these climate differences. Using supplementary lighting can, albeit only partly, counteract the effect of radiation on that climate difference, probably because it changes the light distribution.

The deviation of leaf temperature from canopy air temperature is mainly determined by the radiation components of the energy balance. Therefore, radiation should be the main focus when simulating leaf temperatures. In the context of a further developed canopy, the difference between leaf and canopy air temperature could be decreased by having a lower *LAI* or increasing the temperature of the heating pipe.

## Supporting information

S1 Data (BZ2)

S1 Fig (a) Ambient temperature and (b) canopy air temperature (at 60 cm below the top of the canopy). Bold lines represent the average value, whereas the shaded area represents the standard deviation over the days for sunny (red), cloudy (blue) and partly cloudy days (orange).
(PDF)

S2 Fig (a) Ambient relative humidity and (b) canopy air relative humidity (at 60 cm below the top of the canopy). Bold lines represent the average value, whereas the shaded area represents the standard deviation over the days for sunny (red), cloudy (blue) and partly cloudy days (orange).
(PDF)

S3 Fig Average fraction of the ambient relative humidity (*RH*) and canopy relative humidity (i.e. *RH* of the air 60 cm below the top of the canopy) determined by the temperature effect on the saturated water vapour pressure (thick lines) for sunny (red), cloudy (blue) and partly cloudy days(orange). The shaded area represents the standard deviation. a.) shows the fraction for the course of the day, where the dashed line is the time point of the profile at noon (b), where the dashed lines show the heights at which the fraction between ambient and canopy *RH* explained by addition/removal of water vapour (a) were calculated.
(PDF)

S4 Fig Difference between ambient vapour pressure and canopy vapour pressure (i.e. vapour pressure of the air 60 cm below the top of the canopy). Bold lines represent the average value, whereas the shaded area represents the standard deviation over the days for sunny (red), cloudy (blue) and partly cloudy days (orange). a.) time course of the vapour pressure difference, where the dashed line indicates noon. (b) vertical vapour pressure profile in the canopy at noon; the dashed lines show the heights at which the differences between ambient and canopy vapour pressure were calculated.
(PDF)

S5 Fig Causal path model for the difference between canopy and ambient temperature and absolute humidity that was consistent with the data (p = 0.058, df = 32, Fisher’s C = 45.47), where solid arrows show a causal relation and dashed double-headed arrows indicate correlations without a specified direction. The values denote the standardized path coefficients. The color of the boxes denote the following: white represents the climate variables, grey represents the climate control measures, green represents the crop and orange represents radiation. *Abbreviations: R_outside_ is outside global radiation; R_inside_ is inside global radiation; HPS is High Pressure Sodium lamps, supplemental assimilation lighting; Window is window opening (open or closed); Screen is shading screen position (open or closed); T_pipe_ is temperature of the heating pipe; LAI is leaf area index; T_ambient_ is ambient temperature, temperature above canopy; q_ambient_ is ambient absolute humidity, absolute humidity above canopy*; Δ*T is difference between ambient temperature and air temperature in the canopy*; Δ*q is difference between ambient absolute humidity and absolute humidity in the canopy. Also see* Table 1.
(PDF)

S6 Fig (a) Canopy air temperature and (b) leaf temperature. Bold lines represent the average value, whereas the shaded area represents the standard deviation over the days for sunny (red), cloudy (blue) and partly cloudy days (orange).
(PDF)

S7 Fig Difference between leaf temperature (at 60 cm below the top of the canopy) and ambient temperature. Bold lines represent the average value, whereas the shaded area represents the standard deviation over the days for sunny (red), cloudy (blue) and partly cloudy days (orange). a.) time course of the temperature difference, where the dashed line indicates noon. (b) vertical leaf temperature profile in the canopy at noon; the dashed lines show the heights at which the differences between leaf and ambient temperature were calculated.
(PDF)

S8 Fig Causal path model for the difference between canopy air and leaf temperature that was consistent with the data (p = 0.062, df = 32, Fisher’s C = 45.15), where solid arrows show a causal relation and dashed double-headed arrows indicate correlations without a specified direction. The values denote the standardized path coefficients. The color of the boxes denote the following: white represents the climate variables, grey represents the climate control measures, green represents the crop and orange represents radiation. *Abbreviations: R_outside_ is outside global radiation; R_inside_ is inside global radiation; HPS is High Pressure Sodium lamps, supplemental assimilation lighting; Window is window opening (open or closed); Screen is shading screen position (open or closed); T_pipe_ is temperature of the heating pipe; LAI is leaf area index; T_ambient_ is ambient temperature, temperature above canopy; q_ambient_ is ambient absolute humidity, absolute humidity above canopy*; Δ*T_leaf_ is difference between canopy air temperature and leaf temperature. Also see* Table 1.
(PDF)

S1 Table Harvest dates of the different growing cycles (i.e. from one harvest to the next) with the number of shoots that were measured non-destructively during the growing cycle. (PDF)

S2 Table Days that have been selected for analyzing the difference between the canopy and ambient climate. Crosses indicate if the days are used for the time course and/or path analysis. *LAI* is the *LAI* above the sensor that is 60 cm below the top sensor measuring ambient climate.
(PDF)

S3 Table Output of the path analysis for the difference between ambient and canopy climate. Variables marked with a * are non-directed relations and the coefficients denote the correlation.
(PDF)

S4 Table Output of the path analysis for leaf temperature. Variables marked with a * are non-directed relations and the coefficients denote the correlation.
(PDF)
