# Supplementary material for: Substantial differences occur between canopy and ambient climate: Quantification of interactions in a greenhouse-canopy system
Source: PLoS One. 2020 May 29;15(5):e0233210. doi: 10.1371/journal.pone.0233210 (PMC7259515; doi:10.1371/journal.pone.0233210)
Supplement: S1 Fig — (a) Ambient temperature and (b) canopy air temperature (at 60 cm below the top of the canopy). Bold lines represent the average value, whereas the shaded area represents the standard deviation over the days for sunny (red), cloudy (blue) and partly cloudy days (orange). (PDF) [file pone.0233210.s002.pdf]

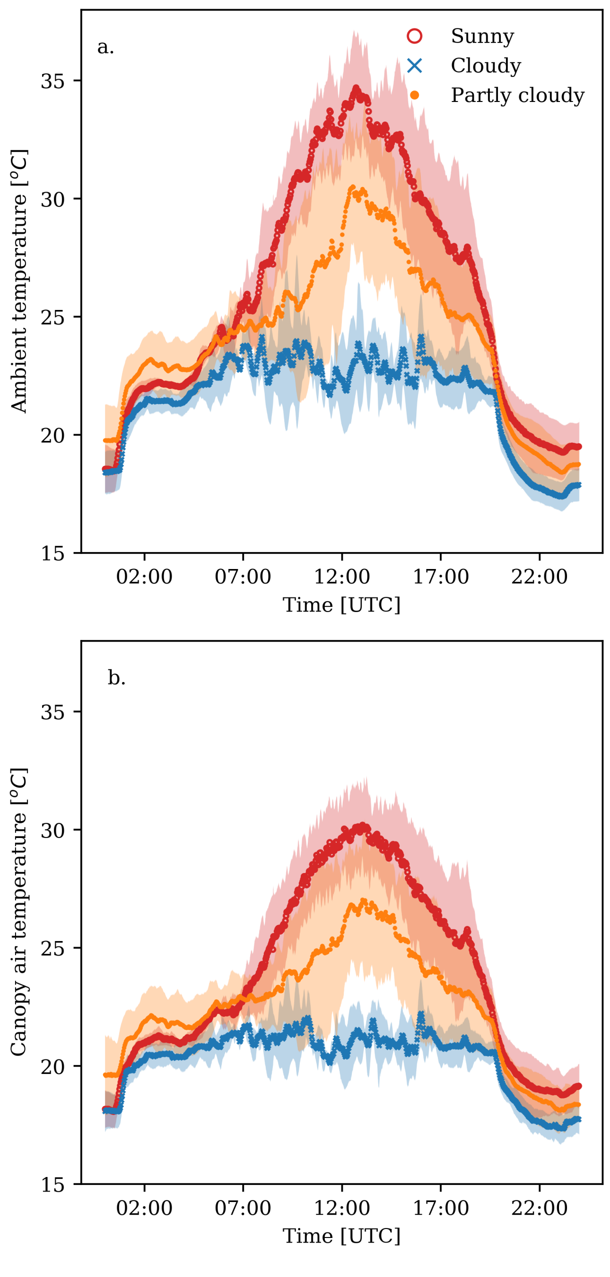

Figure S1: (a) Ambient temperature and (b) canopy air temperature (at 60 cm below the top of the canopy). Bold lines represent the average value, whereas the shaded area represents the standard deviation over the days for sunny (red), cloudy (blue) and partly cloudy days (orange).
