# Supplementary material for: Substantial differences occur between canopy and ambient climate: Quantification of interactions in a greenhouse-canopy system
Source: PLoS One. 2020 May 29;15(5):e0233210. doi: 10.1371/journal.pone.0233210 (PMC7259515; doi:10.1371/journal.pone.0233210)
Supplement: S3 Fig — The shaded area represents the standard deviation. a.) shows the fraction for the course of the day, where the dashed line is the time point of the profile at noon (b), where the dashed lines show the heights at which the fraction between ambient and canopy RH explained by addition/removal of water vapour (a) were calculated. (PDF) [file pone.0233210.s004.pdf]

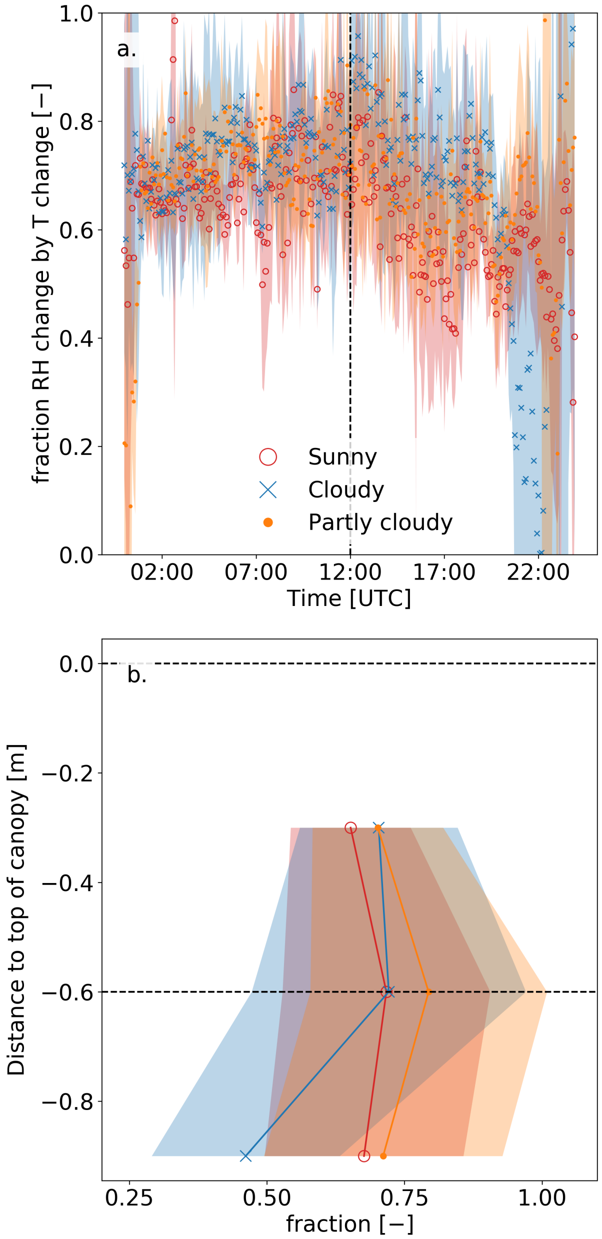

Figure S3: Average fraction of the ambient relative humidity (RH) and canopy relative humidity (i.e. RH of the air 60 cm below the top of the canopy) determined by the temperature effect on the saturated water vapour pressure (thick lines) for sunny (red), cloudy (blue) and partly cloudy days(orange). The shaded area represents the standard deviation. a.) shows the fraction for the course of the day, where the dashed line is the time point of the profile at noon (b), where the dashed lines show the heights at which the fraction between ambient and canopy RH explained by addition/removal of water vapour (a) were calculated.
