# Supplementary material for: Substantial differences occur between canopy and ambient climate: Quantification of interactions in a greenhouse-canopy system
Source: PLoS One. 2020 May 29;15(5):e0233210. doi: 10.1371/journal.pone.0233210 (PMC7259515; doi:10.1371/journal.pone.0233210)
Supplement: S4 Fig — Bold lines represent the average value, whereas the shaded area represents the standard deviation over the days for sunny (red), cloudy (blue) and partly cloudy days (orange). a.) time course of the vapour pressure difference, where the dashed line indicates noon. (b) vertical vapour pressure profile in the canopy at noon; the dashed lines show the heights at which the differences between ambient and canopy vapour pressure were calculated. (PDF) [file pone.0233210.s005.pdf]

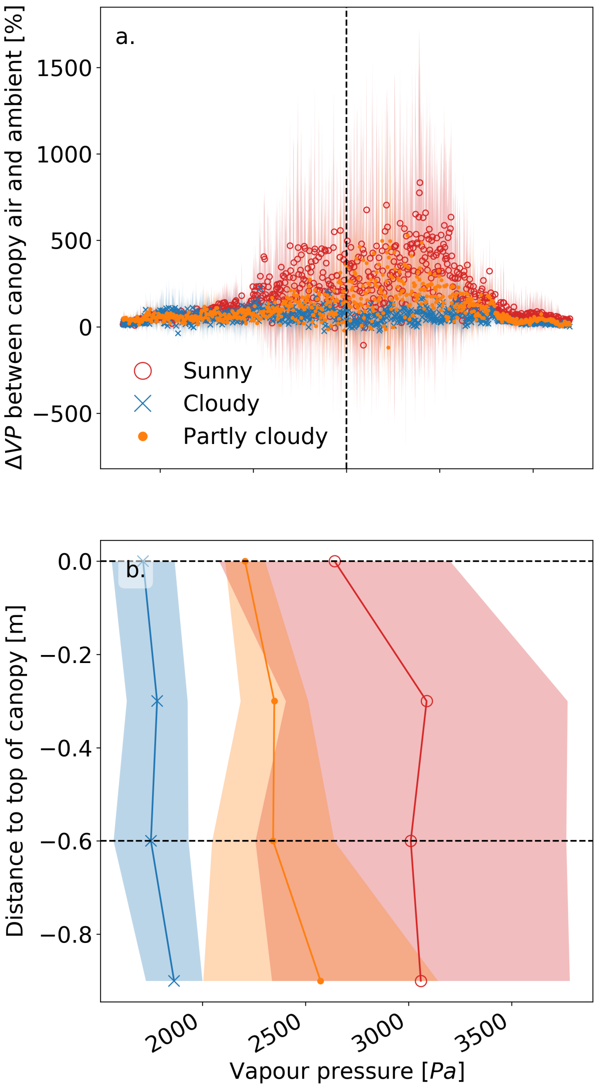

Figure S4: Difference between ambient vapour pressure and canopy vapour pressure (i.e. vapour pressure of the air 60 cm below the top of the canopy). Bold lines represent the average value, whereas the shaded area represents the standard deviation over the days for sunny (red), cloudy (blue) and partly cloudy days (orange). a.) time course of the vapour pressure difference, where the dashed line indicates noon. (b) vertical vapour pressure profile in the canopy at noon; the dashed lines show the heights at which the differences between ambient and canopy vapour pressure were calculated.
