# Supplementary material for: Substantial differences occur between canopy and ambient climate: Quantification of interactions in a greenhouse-canopy system
Source: PLoS One. 2020 May 29;15(5):e0233210. doi: 10.1371/journal.pone.0233210 (PMC7259515; doi:10.1371/journal.pone.0233210)
Supplement: S5 Fig — The values denote the standardized path coefficients. The color of the boxes denote the following: white represents the climate variables, grey represents the climate control measures, green represents the crop and orange represents radiation. Abbreviations: Routside is outside global radiation; Rinside is inside global radiation; HPS is High Pressure Sodium lamps, supplemental assimilation lighting; Window is window opening (open or closed); Screen is shading screen position (open or closed); Tpipe is temperature of the heating pipe; LAI is leaf area index; Tambient is ambient temperature, temperature above canopy; qambient is ambient absolute humidity, absolute humidity above canopy; ΔT is difference between ambient temperature and air temperature in the canopy; Δq is difference between ambient absolute humidity and absolute humidity in the canopy. Also see Table 1. (PDF) [file pone.0233210.s006.pdf]

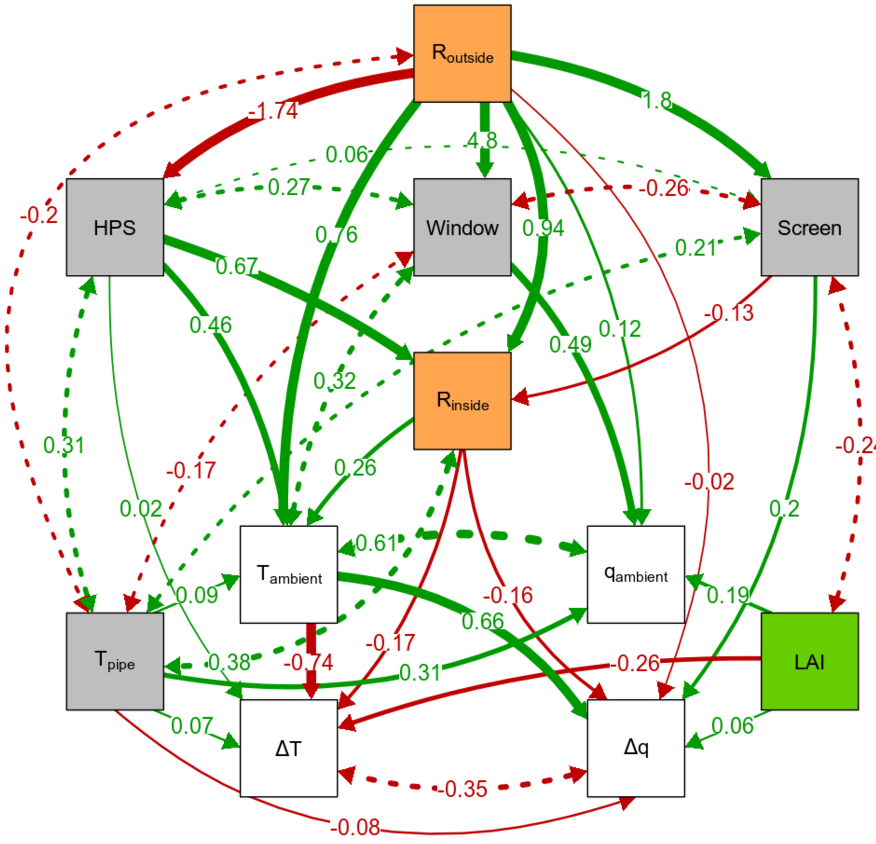

Figure S5: Causal path model for the difference between canopy and ambient temperature and absolute humidity that was consistent with the data (p=0.058, df=32, Fisher's C=45.47), where solid arrows show a causal relation and dashed double-headed arrows indicate correlations without a specified direction. The values denote the standardized path coefficients. The color of the boxes denote the following: *white* represents the climate variables, *grey* represents the climate control measures, *green* represents the crop and *orange* represents radiation. *Abbreviations: $R_{outside}$ is outside global radiation; R_{inside} is inside global radiation; HPS is High Pressure Sodium lamps, supplemental assimilation lighting; Window is window opening (open or closed); Screen is shading screen position (open or closed); T_{pipe} is temperature of the heating pipe; LAI is leaf area index; $T_{ambient}$ is ambient temperature, temperature above canopy; $q_{ambient}$ is ambient absolute humidity, absolute humidity above canopy; ΔT is difference between ambient temperature and air temperature in the canopy; Δq is difference between ambient absolute humidity and absolute humidity in the canopy. Also see Table 1.*
