# Supplementary material for: Substantial differences occur between canopy and ambient climate: Quantification of interactions in a greenhouse-canopy system
Source: PLoS One. 2020 May 29;15(5):e0233210. doi: 10.1371/journal.pone.0233210 (PMC7259515; doi:10.1371/journal.pone.0233210)
Supplement: S7 Fig — Bold lines represent the average value, whereas the shaded area represents the standard deviation over the days for sunny (red), cloudy (blue) and partly cloudy days (orange). a.) time course of the temperature difference, where the dashed line indicates noon. (b) vertical leaf temperature profile in the canopy at noon; the dashed lines show the heights at which the differences between leaf and ambient temperature were calculated. (PDF) [file pone.0233210.s008.pdf]

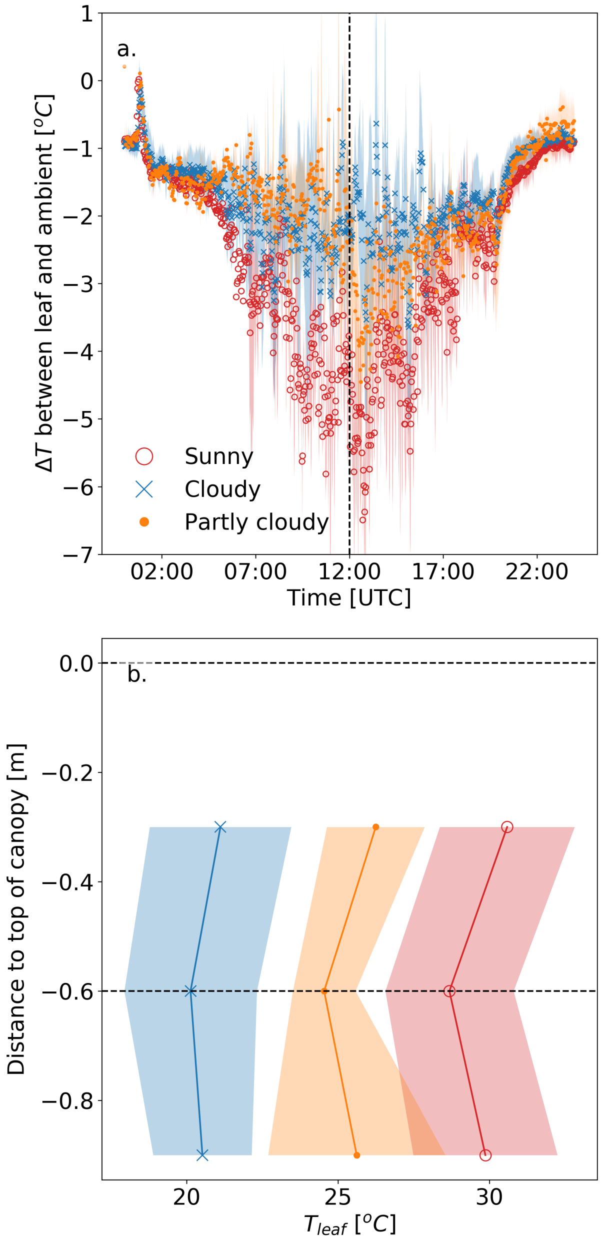

Figure S7: Difference between leaf temperature (at 60 cm below the top of the canopy) and ambient temperature. Bold lines represent the average value, whereas the shaded area represents the standard deviation over the days for sunny (red), cloudy (blue) and partly cloudy days (orange). a.) time course of the temperature difference, where the dashed line indicates noon. (b) vertical leaf temperature profile in the canopy at noon; the dashed lines show the heights at which the differences between leaf and ambient temperature were calculated.
