# Supplementary material for: Substantial differences occur between canopy and ambient climate: Quantification of interactions in a greenhouse-canopy system
Source: PLoS One. 2020 May 29;15(5):e0233210. doi: 10.1371/journal.pone.0233210 (PMC7259515; doi:10.1371/journal.pone.0233210)
Supplement: S1 Table — (PDF) [file pone.0233210.s010.pdf]

Supplementary Material

Table S1: Harvest dates of the different growing cycles (i.e. from one harvest to the next) with the number of shoots that were measured non-destructively during the growing cycle.

Cycle	Harvest date [dd-mm-yyyy]	Nr. shoots [#]
1	06-12-2016	50
2	26-01-2017	16
3	16-03-2017	16
4	02-05-2017	24
5	14-06-2017	12
