# Supplementary material for: Substantial differences occur between canopy and ambient climate: Quantification of interactions in a greenhouse-canopy system
Source: PLoS One. 2020 May 29;15(5):e0233210. doi: 10.1371/journal.pone.0233210 (PMC7259515; doi:10.1371/journal.pone.0233210)
Supplement: S2 Table — Crosses indicate if the days are used for the time course and/or path analysis. LAI is the LAI above the sensor that is 60 cm below the top sensor measuring ambient climate. (PDF) [file pone.0233210.s011.pdf]

Table S2: Days that have been selected for analyzing the difference between the canopy and ambient climate. Crosses indicate if the days are used for the time course and/or path analysis. *LAI* is the *LAI* above the sensor that is 60 cm below the top sensor measuring ambient climate.

Date	Time course	path analysis	type	*LAI*
06-05-2017		x	Sunny	0.00
09-05-2017		x	Partly cloudy	0.00
12-05-2017		x	Cloudy	0.00
15-05-2017		x	Sunny	0.00
19-05-2017		x	Cloudy	0.01
21-05-2017		x	Sunny	0.03
24-05-2017		x	Partly cloudy	0.14
25-05-2017	x		Sunny	0.22
26-05-2017	x	x	Sunny	0.33
27-05-2017	x		Sunny	0.46
28-05-2017	x		Partly cloudy	0.59
30-05-2017	x	x	Partly cloudy	0.80
31-05-2017		x	Partly cloudy	0.86
01-06-2017	x		Sunny	0.91
02-06-2017	x	x	Sunny	0.93
06-06-2017	x	x	Cloudy	0.97
07-06-2017	x	x	Cloudy	0.97
08-06-2017	x		Partly cloudy	0.97
09-06-2017	x	x	Partly cloudy	0.97
11-06-2017		x	Sunny	0.97
12-06-2017	x	x	Cloudy	0.97
13-06-2017	x		Cloudy	0.97
