# Supplementary material for: Substantial differences occur between canopy and ambient climate: Quantification of interactions in a greenhouse-canopy system
Source: PLoS One. 2020 May 29;15(5):e0233210. doi: 10.1371/journal.pone.0233210 (PMC7259515; doi:10.1371/journal.pone.0233210)
Supplement: S3 Table — Variables marked with a * are non-directed relations and the coefficients denote the correlation. (PDF) [file pone.0233210.s012.pdf]

Table S3: Output of the path analysis for the difference between ambient and canopy climate. Variables marked with a * are non-directed relations and the coefficients denote the correlation.

Response	Independent param.	coefficient	Norm. coefficient	p-value
$q_{ambient}$	LAI	0.33968	0.193	0.000
$q_{ambient}$	T_{pipe}	0.19389	0.309	0.000
$q_{ambient}$	$R_{outside}$	0.00064	0.115	0.058
$q_{ambient}$	Window	0.89793	0.494	0.000
$T_{ambient}$	HPS	1.65298	0.462	0.000
$T_{ambient}$	R_{inside}	0.01243	0.258	0.000
$T_{ambient}$	$R_{outside}$	0.00832	0.760	0.000
$T_{ambient}$	T_{pipe}	0.10689	0.087	0.001
ΔT	$T_{ambient}$	-0.16197	-0.736	0.000
ΔT	LAI	-0.15274	-0.256	0.000
ΔT	R_{inside}	-0.00230	-0.171	0.000
ΔT	HPS	0.04223	0.021	0.280
ΔT	T_{pipe}	0.01219	0.072	0.042
Δq	LAI	0.03093	0.063	0.003
Δq	R_{inside}	-0.00108	-0.158	0.000
Δq	$R_{outside}$	-0.00003	-0.023	0.685
Δq	$T_{ambient}$	0.09337	0.658	0.000
Δq	T_{pipe}	-0.01395	-0.080	0.000
Δq	Screen	0.10386	0.205	0.000
R_{inside}	$R_{outside}$	0.21470	0.944	0.000
R_{inside}	HPS	49.52828	0.667	0.000
R_{inside}	Screen	-9.81450	-0.132	0.039
Window	$R_{outside}$	0.01469	4.796	0.000
Screen	$R_{outside}$	0.00552	1.803	0.000
HPS	$R_{outside}$	-0.00533	-1.741	0.000
ΔT^*	Δq^*	-0.34830	-0.348	0.000
$T^*_{ambient}$	$q^*_{ambient}$	0.60700	0.607	0.000
$T_{ambient}$	Window	0.32450	0.324	0.000
T^*_{pipe}	$R^*_{outside}$	-0.20410	-0.204	0.000
T^*_{pipe}	R^*_{inside}	0.37590	0.376	0.000
Screen*	LAI*	-0.24380	-0.244	0.000
Screen*	T^*_{pipe}	0.21380	0.214	0.000
Screen*	HPS*	0.01710	0.017	0.382
Window*	T^*_{pipe}	-0.17190	-0.172	0.001
Window*	Screen*	-0.25590	-0.256	0.000
Window*	HPS*	0.24630	0.246	0.000
T^*_{pipe}	HPS*	0.35680	0.357	0.000
