# Supplementary material for: Substantial differences occur between canopy and ambient climate: Quantification of interactions in a greenhouse-canopy system
Source: PLoS One. 2020 May 29;15(5):e0233210. doi: 10.1371/journal.pone.0233210 (PMC7259515; doi:10.1371/journal.pone.0233210)
Supplement: S4 Table — Variables marked with a * are non-directed relations and the coefficients denote the correlation. (PDF) [file pone.0233210.s013.pdf]

Table S4: Output of the path analysis for leaf temperature. Variables marked with a * are non-directed relations and the coefficients denote the correlation.

Response	Independent param.	coefficient	Norm. coefficient	p-value
ΔT_{leaf}	LAI	1.18315	0.501	0.000
ΔT_{leaf}	R_{inside}	-0.00180	-0.250	0.000
ΔT_{leaf}	$R_{outside}$	0.00051	0.328	0.000
ΔT_{leaf}	HPS	0.12609	0.030	0.006
ΔT_{leaf}	T_{pipe}	-0.05753	-0.336	0.000
T_{canopy}	HPS	1.10446	0.391	0.000
T_{canopy}	R_{inside}	0.00695	0.185	0.002
T_{canopy}	$R_{outside}$	0.00488	0.483	0.000
T_{canopy}	T_{pipe}	0.17430	0.170	0.000
T_{canopy}	Screen	2.06099	0.729	0.004
T_{canopy}	LAI	-4.03524	-0.217	0.000
q_{canopy}	LAI	-0.00330	-0.216	0.000
q_{canopy}	T_{pipe}	0.00026	0.305	0.000
q_{canopy}	R_{inside}	0.00000	0.135	0.009
q_{canopy}	Screen	0.00292	1.257	0.000
R_{inside}	$R_{outside}$	0.29247	1.089	0.000
R_{inside}	HPS	44.05805	0.587	0.000
R_{inside}	Screen	-95.37331	-1.270	0.000
HPS	$R_{outside}$	-0.00504	-1.410	0.000
Screen	$R_{outside}$	0.01214	3.396	0.000
Window	$R_{outside}$	0.00995	2.783	0.001
T^*_{canopy}	Window*	0.22000	0.220	0.006
T^*_{canopy}	q^*_{canopy}	0.76160	0.762	0.000
HPS*	Window*	0.39440	0.394	0.000
T^*_{pipe}	R^*_{inside}	0.40020	0.400	0.000
T^*_{pipe}	HPS*	0.22080	0.221	0.006
